# Breaking the tetrahedral constraint of tetracoordination: Square-planar [ICl_4_] enables unprecedented birefringence in [C(NH_2_)_3_][ICl_4_]

**DOI:** 10.1126/sciadv.aef7948

**Published:** 2026-10-16

**Authors:** Zhiwei Sun, Qingran Ding, Weiqi Huang, Peixuan Zhu, Guimei Lai, Xue Mei, Sangen Zhao, Junhua Luo

**Affiliations:** ^1^ College of Chemistry and Materials, Jiangxi Normal University, Nanchang, Jiangxi, 330022, P. R. China.; ^2^ State Key Laboratory of Functional Crystals and Devices, Chinese Academy of Sciences Fujian Institute of Research on the Structure of Matter, Fuzhou, Fujian, 350002, P. R. China.; ^3^ Quantum Science Center of Guangdong-Hong Kong-Macao Greater Bay Area, Shenzhen, Guangdong, 518000, P. R. China.

## Abstract

Birefringent crystals are indispensable for optoelectronic applications, yet commercially available crystals still suffer from the drawback of insufficient birefringence. Meanwhile, tetracoordination units—traditionally confined to a tetrahedral geometry—have long been sidelined from mainstream high-performance birefringent materials due to their intrinsic nearly negligible polarizability anisotropy. Herein, we break this tetrahedral constraint—demonstrating tetracoordinate square-planar [ICl_4_] anions as a revolutionary platform for unprecedented birefringence. To achieve this, we report an improved synthesis method that avoids the use of toxic Cl_2_ gas, enabling the preparation of [C(NH_2_)_3_][ICl_4_] featuring square-planar [ICl_4_] units. This compound displays a birefringence of 0.975@550 nm, setting a new record that surpasses all existing compounds containing tetracoordinate structural units. The giant birefringence of this material is attributed to the strong polarization anisotropy induced by the unique square-planar geometry of the [ICl_4_] tetracoordinate units. This research provides theoretical guidance for designing high-performance polar materials based on tetracoordinate units in giant birefringent materials and promotes applications in related optoelectronic device.

## INTRODUCTION

Birefringent crystals, as core functional materials for optical modulation, play an irreplaceable role in precision optoelectronic devices including optical isolators, waveplates, and electro-optic modulators. (*1*–*5*) The birefringent effect serves as the physical foundation for realizing critical functions such as optical signal phase modulation, polarization separation, and optical path control. (*6*–*8*) Driven by these functional requirements, commercial crystals exhibit significant performance limitations in practical applications. From birefringence parameters, LiNbO_3_ (Δ*n* = 0.076@1064 nm) (*9*) and MgF_2_ (Δ*n* = 0.012@560 nm) (*10*) exhibit low values (< 0.1), merely supporting basic polarized beam splitting. Although α-BaB_2_O_4_ (α-BBO) (Δ*n* = 0.120@1064 nm) (*11*) CaCO_3_ (Δ*n* = 0.171@630 nm) (*12*), YVO_4_ (Δ*n* = 0.208@1064 nm) (*13*), and TiO_2_ (Δ*n* = 0.260@1300 nm) (*14*) exhibit relatively higher birefringence values, their values remain below 0.3, hindering their application in miniaturized and integrated optical devices. (*15*)

To overcome the performance limitations of current birefringent materials, the primary strategy for enhancing birefringence lies in increasing the polarizability anisotropy of molecular. (*16*, *17*) From a molecular design viewpoint, current research mainly focuses on constructing π-conjugated molecular frameworks with highly anisotropic electron distribution to achieve this. (*18*–*21*) Linear π-conjugated units (e.g., C≡N, C=S) and large planar aromatic rings {e.g., [C_3_N_3_O_3_] (*22*), [C_3_N_6_] (*23*–*25*), [C_6_N_9_] (*26*), [C_9_N_13_] (*27*)} can significantly enhance the in-plane polarizability along the conjugation direction (α_⊥_) through molecular orbital delocalization. The out-of-plane polarizability (α_⊥_) remains unaffected, thereby effectively increasing the polarizability anisotropy (Δα = α_⊥_ − α_⊥_). Recently, linear halogen anions ([I_3_] (*15*), [ICl_2_] (*28*), [IBr_2_] (*28*)) can selectively enhance the polarizability anisotropy (Δα) along the molecular axis, making them ideal structural building blocks for constructing birefringent materials. Thus, exploring building blocks with high structural anisotropy (including π-conjugated aromatic rings, linear chains, polar pyramids, twisted polyhedral) has emerged as an effective strategy for enhancing birefringence properties. (*29*)

However, non-π-conjugated tetracoordinate building blocks have been gradually excluded from conventional mainstream design schemes of birefringent compounds. The primary reason lies in the fact that tetracoordinate units predominantly adopt tetrahedral configurations with *T_d_* symmetry (Fig. 1A), leading to nearly negligible polarizability anisotropy (Δα ≈ 0) in such structural motifs. (*30*, *31*) To achieve enhanced anisotropy, the partial substitution strategy has been extensively adopted, enabling the introduction of heteroatoms or functional groups (F, S, N, OH, NH_2_) into tetracoordinate motifs. This strategy has successfully developed numerous new building units such as [BX_4_], [SiX_4_], [PX_4_], and [SX_4_] (X = F, S, N, OH, NH_2_, depicted in Fig. 1B and table S1) (*21*). Nevertheless, the compounds constructed from these motifs still face significant challenges in birefringence enhancement — their birefringence indices (Δ*n*) commonly lie between 0.05 and 0.30. Only a few exceptional compounds exhibit birefringence values over 0.3. Substitution-based examples include K(3-C_5_H_4_NSO_3_) (Δ*n* = 0.312@546 nm) (*32*), C_3_N_6_H_7_SO_3_NH_2_ (Δ*n* = 0.340@546 nm) (*25*, *33*), and (C_3_N_6_H_7_)SO_3_CH_3_·H_2_O (Δ*n* = 0.310@546 nm) (*24*). Rare examples of giant birefringence reported via other strategies include Hg_4_(Te_2_O_5_)(SO_4_) (Δ*n* = 0.542@546 nm) (*33*). This implies that traditional strategies relying on these tetrahedral coordinate units are perhaps approaching their performance limits, with limited room for further enhancement.

**Fig. 1.**
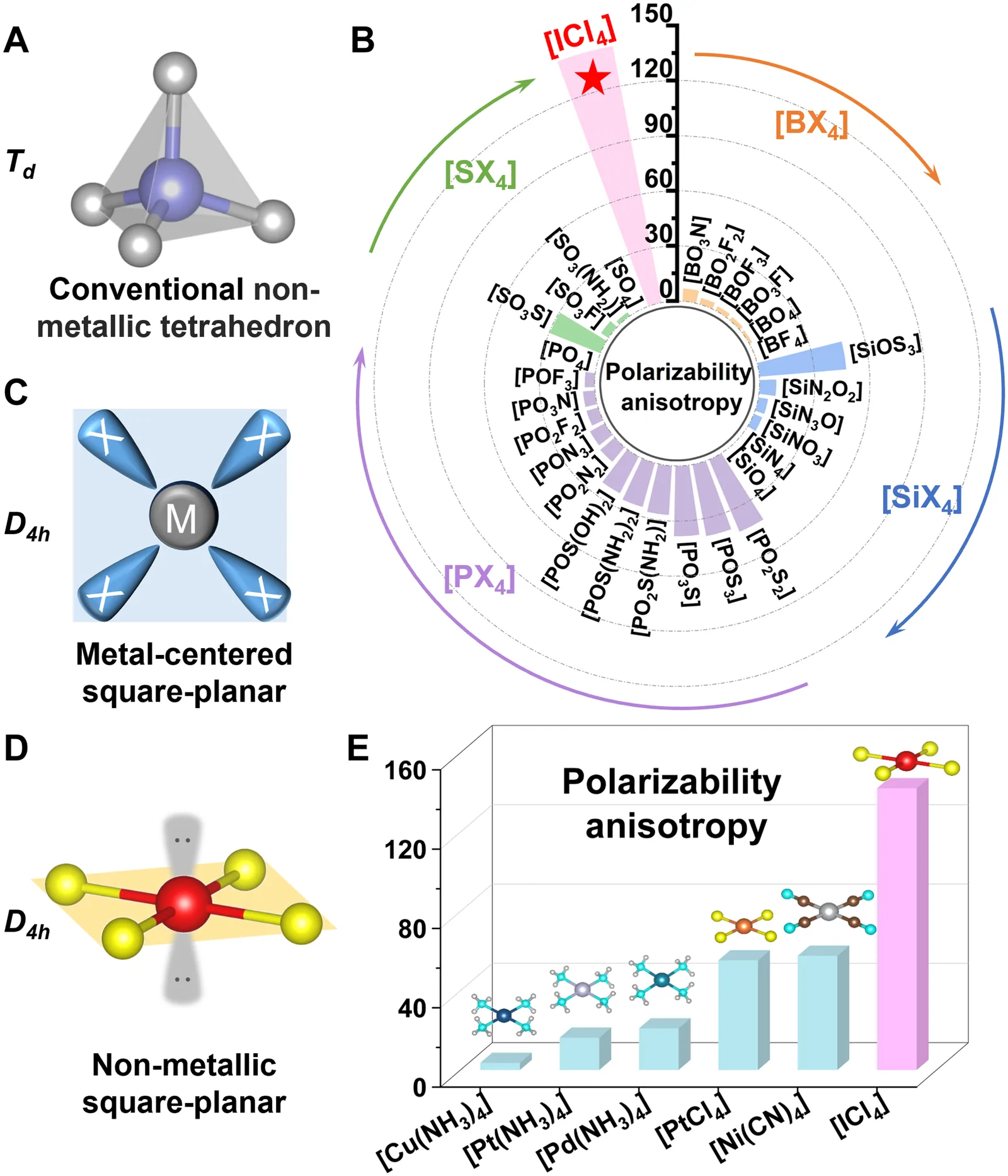
Comparison of the structural and polarizability anisotropy between the traditional tetracoordination unit and [ICl_4_] unit. (**A**) Traditional non-metallic tetrahedral unit (*T_d_* symmetry). (**B**) Comparison of the polarizability anisotropy between the traditional non-metallic tetrahedral units and the [ICl_4_] unit. (**C**) Metal-centered square-planar unit (*D_4h_* symmetry). (**D**) Non-metallic square-planar unit (*D_4h_* symmetry). (**E**) Comparison of the polarizability anisotropy between the metal-centered square-planar units and the [ICl_4_] unit.

Here, we propose a strategy: within the fixed tetracoordination (CN = 4), enhancing the polarizability anisotropy directly via spatial configuration regulation, rather than chemical composition modification. Focusing on the square-planar coordination unit with *D_4h_* symmetry (metal-centered square-planar units, shown in Fig. 1C), the coplanar arrangement of atoms within this geometry substantially increased the disparity in polarizability between in-plane and out-of-plane directions. Screening of square-planar structural units via first-principles calculations showed that the nonmetallic square-planar [ICl_4_] (Fig. 1D) exhibits the highest polarizability anisotropy, with Δα reaching 142.1 (Fig. 1E and table S2). In this work, the compound [C(NH_2_)_3_][ICl_4_] featuring the [ICl_4_] units with high polarizability anisotropy was successfully synthesized via a green and facile aqueous solution method. This compound exhibits an exceptional birefringence of 0.975 at 550 nm, significantly surpassing that of previously reported compounds with tetracoordinate structural units. The giant birefringence is driven by the square-planar tetracoordinate [ICl_4_] unit. This work not only offers a new perspective for studying coordination structure-property relationships but also paves the way for designing high-performance optical devices through coordination geometry innovation.

## RESULTS

How can [ICl_4_]-based compounds be efficiently constructed? Systematic investigations reveal that conventional synthesis methods for these materials all require the use of toxic chlorine gas (Cl_2_) (Fig. 2A). Typical examples include A[ICl_4_] (A = Na, K, Rb, Cs inorganic cations) (*34*) and B[ICl_4_] (B = various organic cations) (*35*). Additionally, energy change calculations for the synthesis of Cs[ICl_4_] (as depicted in Fig. 2B) reveal that both reaction steps—from CsCl to the intermediate Cs[ICl_2_] and then to the target Cs[ICl_4_]—exhibit exothermic reaction with low activation energy barriers to overcome. Thus, the reaction system possesses both thermodynamic spontaneity—indicated by negative Gibbs free energy change—and kinetic accessibility, enabling its spontaneous occurrence under mild conditions. (*36*) Based on this, it is speculated that the synthesis of [ICl_4_]-based compounds may not require the use of strong oxidizing agent Cl_2_ as a reaction condition. This deduction prompted us to further pursue research on novel synthetic methodologies. Inspired by this, this work successfully prepared the [ICl_4_]-based compound [C(NH_2_)_3_][ICl_4_] without Cl_2_ involvement. A facile aqueous solution method consistent with green chemistry principles was adopted (Fig. 2A): the reaction of guanidine hydrochloride with iodine(V) oxide in a strongly acidic solution (pH < 1) yielded the desired compound.

**Fig. 2.**
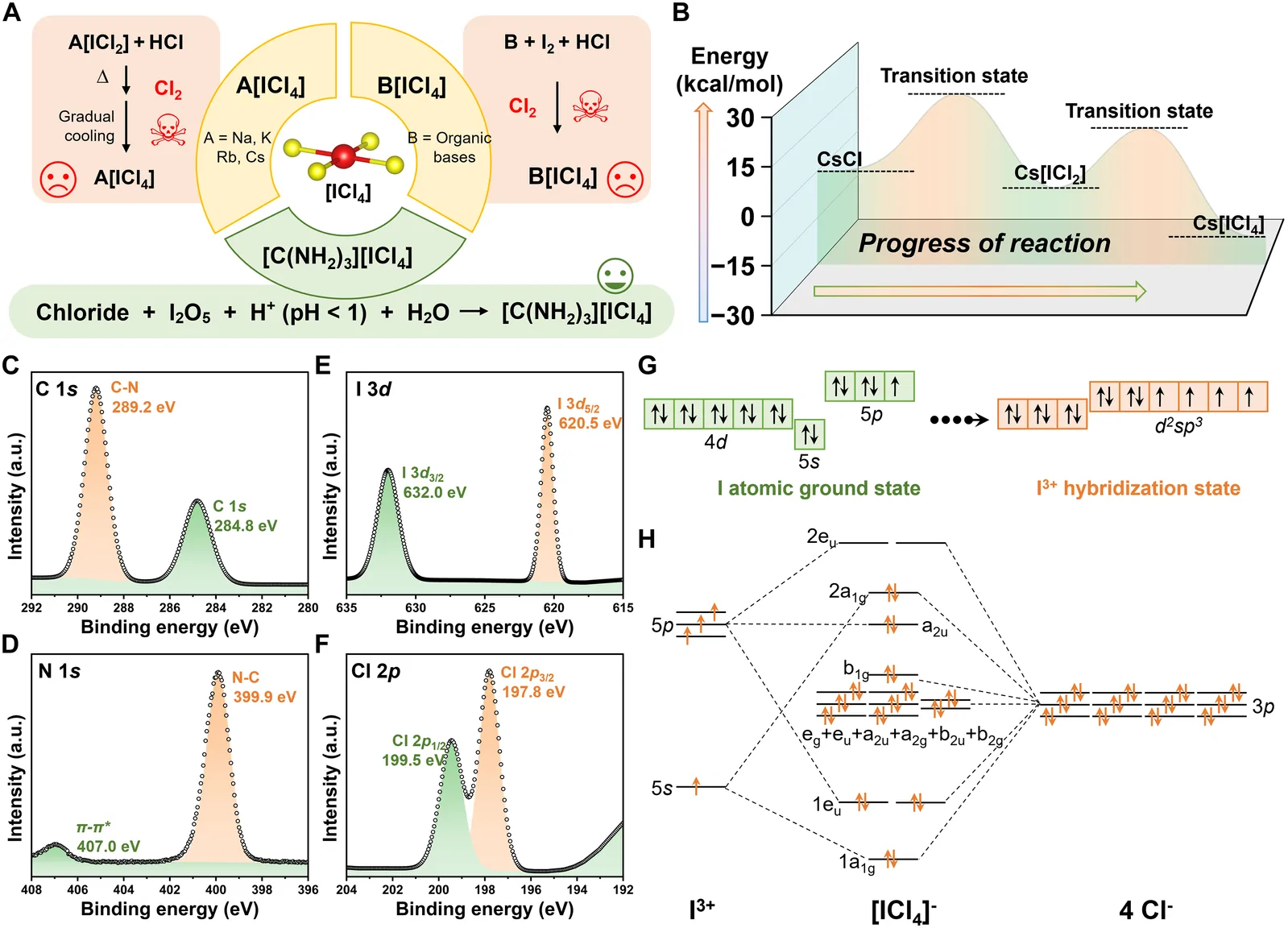
Synthesis characterization and electronic structure of [ICl_4_] unit. (**A**) Synthesis methods related to [ICl_4_] unit. (**B**) Reaction pathway from CsCl to Cs[ICl_2_] to Cs[ICl_4_]. (**C**) C 1*s*, (**D**) N 1*s*, (**E**) I 3*d*, and (**F**) Cl 2*p* XPS spectra of compound [C(NH_2_)_3_][ICl_4_]. (**G**) Hybridization orbital formation process of the central iodine (I) atom in [ICl_4_] unit. (**H**) Molecular orbital energy levels diagram of the [ICl_4_] unit.

The x-ray photoelectron spectroscopy (XPS) measurements were conducted to investigate the valence states of elements and their bonding environments in the compound [C(NH_2_)_3_][ICl_4_]. The binding energies were calibrated against the C 1s peak at 284.8 eV. As shown in Fig. 2C, another peak of C 1*s* at 289.2 eV corresponds to the characteristic peak of the C-N single bond. For the N 1*s* spectrum shown in Fig. 2D, the primary peak at 399.9 eV is assigned to C−N single bonds. A second peak at 407.0 eV, characterized by higher binding energy and lower intensity, is attributed to π-π* interactions (*37*, *38*) of [C(NH_2_)_3_] units. Relevant literature data indicate that the I *d*_5/2_ peaks for iodine in oxidation states −1, 0, and +5 are located at 618.2–619.7 eV, 619.9 eV, and 623.3 eV, respectively. Given the absence of standard spectral references for I^3+^ in databases, the I 3*d*_5/2_ peak of iodine in the +3 intermediate oxidation state within [ICl_4_] is inferred to lie between 619.9 eV (0 valence) and 623.3 eV (+5 valence). As shown in Fig. 2E, this peak is experimentally observed at 620.5 eV, consistent with the predicted interval. The Cl 2*p*_3/2_ peak at 197.8 eV in Fig. 2F is characteristic of chlorine in the −1 valence. Spectroscopic information of C-N bond formation and the valence states of I/Cl is in excellent agreement with the structural information derived from single-crystal x-ray analysis.

For the [ICl_4_] units, the molecular geometry and its bond formation mechanism will be systematically explored via valence-shell electron-pair repulsion (VSEPR) theory, hybrid orbital theory and molecular orbital (MO) theory. The I (III) central atom exhibits *d^2^sp^3^* hybridization (Fig. 2G), where four *d^2^sp^3^* hybrid orbitals form σ bonds with Cl ions, and the remaining two hybrid orbitals are occupied by lone-pair electrons. This bond formation and two lone-pair electrons distribution predict a square-planar geometry with *D_4h_* symmetry, consistent with the VSEPR theory. To further investigate the orbital energy levels and bonding pattern of [ICl_4_], a molecular orbital energy level diagram (Fig. 2H) was constructed, considering only the participation of I 5*s*/5*p* and Cl 3*p* orbitals in bonding interactions. The 4*d* orbitals of the central iodine atom were intentionally omitted as they contribute negligibly to the bonding in this system, a simplification intended to clarify the principal bonding features. This treatment is clearly distinguished from the hybridization model presented in Fig. 2G, where the introduction of *d*-orbitals is required to fully describe the coordination geometry. The 5*s* orbital of central I splits significantly due to ligand-field interactions, with the σ* antibonding orbital (2a_1g_), formed by the symmetric match of 5*s*(I) and 3*p*(Cl), occupying a high-energy position. Consequently, all valence electrons occupy lower-energy bonding or nonbonding orbitals in a paired manner, establishing a low-spin state. This result further confirms that the [ICl_4_] anion exhibit a square-planar configuration with *D_4h_* symmetry.

The crystal structure of [C(NH_2_)_3_][ICl_4_] was successfully determined through single-crystal x-ray diffraction (XRD) analysis. The detailed data are provided in tables S3 to S7 in the Supporting Information. As shown in fig. S1, the experimental powder XRD pattern of [C(NH_2_)_3_][ICl_4_] is in good agreement with the calculated powder XRD pattern based on the single crystal structure, confirming the phase purity of the compound. Thermal stability analysis indicates that [C(NH_2_)_3_][ICl_4_] remains stable up to 398 K (fig. S2), with continuous weight loss observed thereafter. The energy dispersive x-ray (EDX) spectroscopy mapping (fig. S3) shows the existence of C, N, I, and Cl elements with uniform distribution in the [C(NH_2_)_3_][ICl_4_]. The UV-Vis-NIR diffuse reflectance spectrum (fig. S4) reveals an absorption edge at approximately 448 nm. According to Kubelka–Munk function, the reflectance spectra were converted to yield the experimental energy gap of 2.34 eV.

The compound [C(NH_2_)_3_][ICl_4_] crystallizes in the monoclinic crystal system of space group *P*2_1_/*c*. In an asymmetric unit, crystallographically independent atoms include one C atom, three N atoms, two I atoms (I1 and I2), four Cl atoms, and six H atoms. One C atom bonds to three N atoms, and each N atom further connects to two H atoms to form [C(NH_2_)_3_] (Fig. 3A). The I1 atom coordinates with two Cl atoms to form [I1Cl_4_] with bond lengths of 2.4918(14) Å and 2.4982(13) Å. Similarly, I2 atom coordinates with another two Cl atoms to generate [I2Cl_4_], with bond lengths recorded as 2.4956(14) Å and 2.4983(13) Å. The differences in bond lengths among the four I-Cl bonds are no more than 0.0065 Å. This indicates the equivalence of these I-Cl bonds, thus forming a square-planar [ICl_4_] unit (Fig. 3B). The [ICl_4_] and [C(NH_2_)_3_] ions further establish a zero-dimensional (0D) framework via ionic bonding interactions (Fig. 3C), and stack in parallel along the crystallographic *b*-axis direction (Fig. 3D). The square-planar [ICl_4_] units form angles of 26.4° (Fig. 3E) and 26.8° (Fig. 3F) with the crystallographic *ac*-plane, corresponding to angles of 63.6° and 63.2° with the *b*-axis; the planar triangular [C(NH_2_)_3_] units form a 25.4° angle (Fig. 3G) with the crystallographic *ac*-plane, corresponding to a 64.6° angle with the *b*-axis. The specific angle data for [C(NH_2_)_3_] and [ICl_4_] units relative to the different reference systems in this compound are listed in table S8. In Fig. 3, E to G, the orientation angles are defined with respect to the crystallographic planes, whereas the corresponding angles provided in table S8 are defined relative to the crystallographic axes. These two angular definitions are complementary (i.e., their sum equals 90°), thus describing the same geometric arrangement in two equivalent ways. This correspondence has been further illustrated schematically in fig. S5. Notably, both types of units exhibit the largest angles with the *b*-axis among the three axes.

**Fig. 3.**
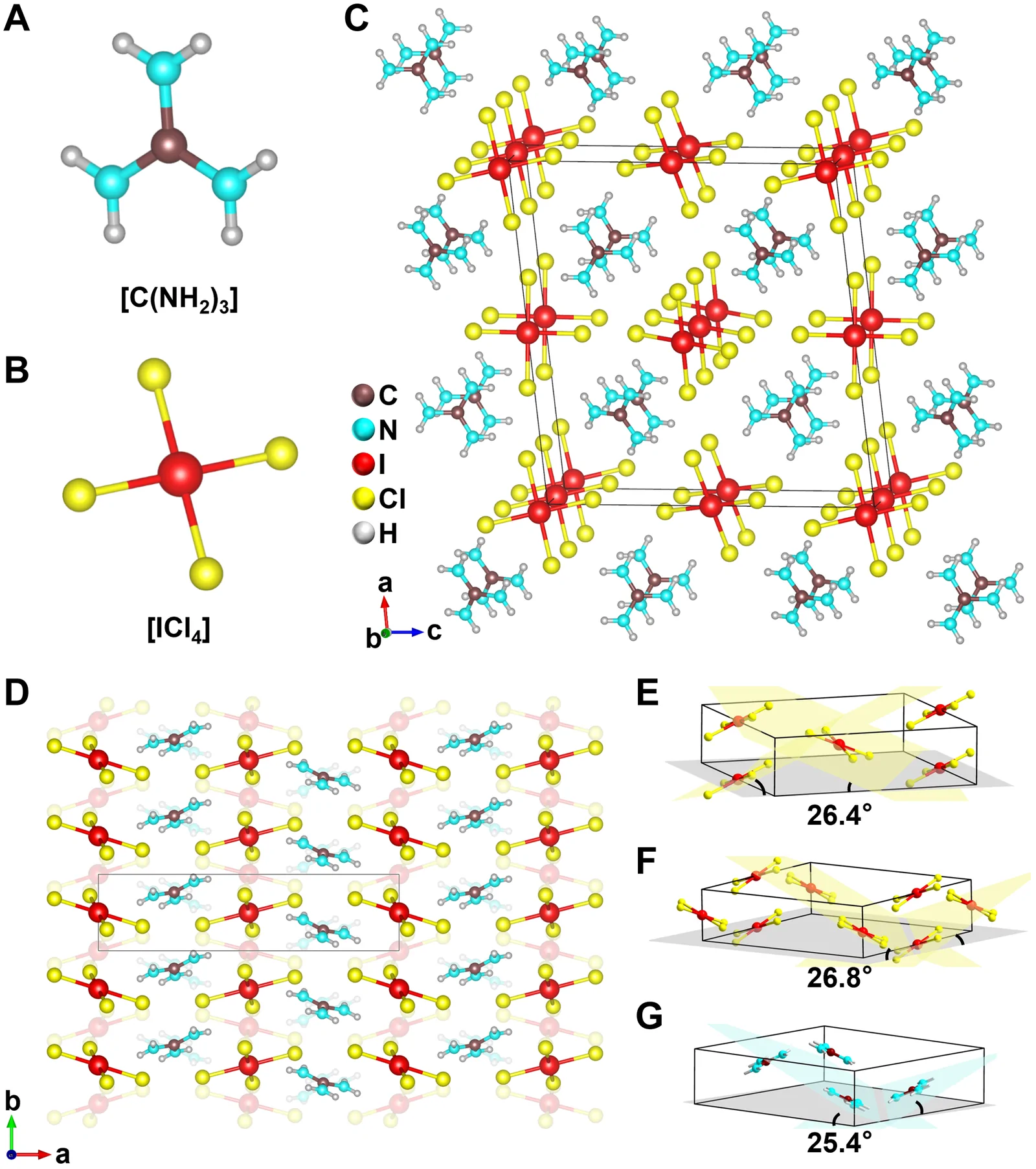
The structure of compound [C(NH_2_)_3_][ICl_4_]. (**A**) The cationic unit [C(NH_2_)_3_]. (**B**) The anionic unit [ICl_4_]. (**C**) The crystal structure of [C(NH_2_)_3_][ICl_4_] observed along the *b*-axis. (**D**) The crystal structure of [C(NH_2_)_3_][ICl_4_] observed along the *c*-axis. (**E**) and (**F**) The angle between [ICl_4_] and *ac*-plane. (**G**) The angle between [C(NH_2_)_3_] and *ac*-plane.

The birefringence properties of [C(NH_2_)_3_][ICl_4_] crystals were characterized using a Nikon LV100N POL polarizing microscope equipped with a Berek compensator. The schematic diagram of the polarizing microscope testing device is shown in Fig. 4A. Under polarized light, the [C(NH_2_)_3_][ICl_4_] crystal displays interference phenomena, which are presented in Fig. 4B. Complete extinction was achieved by adjusting the Berek compensator using both positive and negative compensation modes, as shown in Fig. 4C and 4D, respectively. To clearly distinguish between these two compensation settings and prevent any potential confusion, the corresponding raw polarized light microscopy images have been provided in fig. S6 of the Supporting Information. The optical path difference of the crystal at 550 nm was determined to be 1063 nm via precise reading of the compensator. Simultaneously, the optical microscope was employed for thickness measurement of the crystal sample, yielding an actual thickness of 1.09 μm (Fig. 4E). Using *R* = Δ*n* × *d*, where *R* is the optical path difference, *d* is the thickness, and Δ*n* is the birefringence, the birefringence of [C(NH_2_)_3_][ICl_4_] at 550 nm is determined to be 0.975. The (001) crystallographic plane of the test sample was determined to be parallel to the *ab*-plane by single-crystal x-ray diffraction (Fig. 4F). Theoretical calculations further support this finding, showing a calculated birefringence value of 0.954 (*n* [100] – *n* [010]) at 550 nm for the compound (Fig. 4G and fig. S7). The excellent agreement between experimental and calculated values strongly validates the reliability of the structural model and the accuracy of the measurement methodology.

**Fig. 4.**
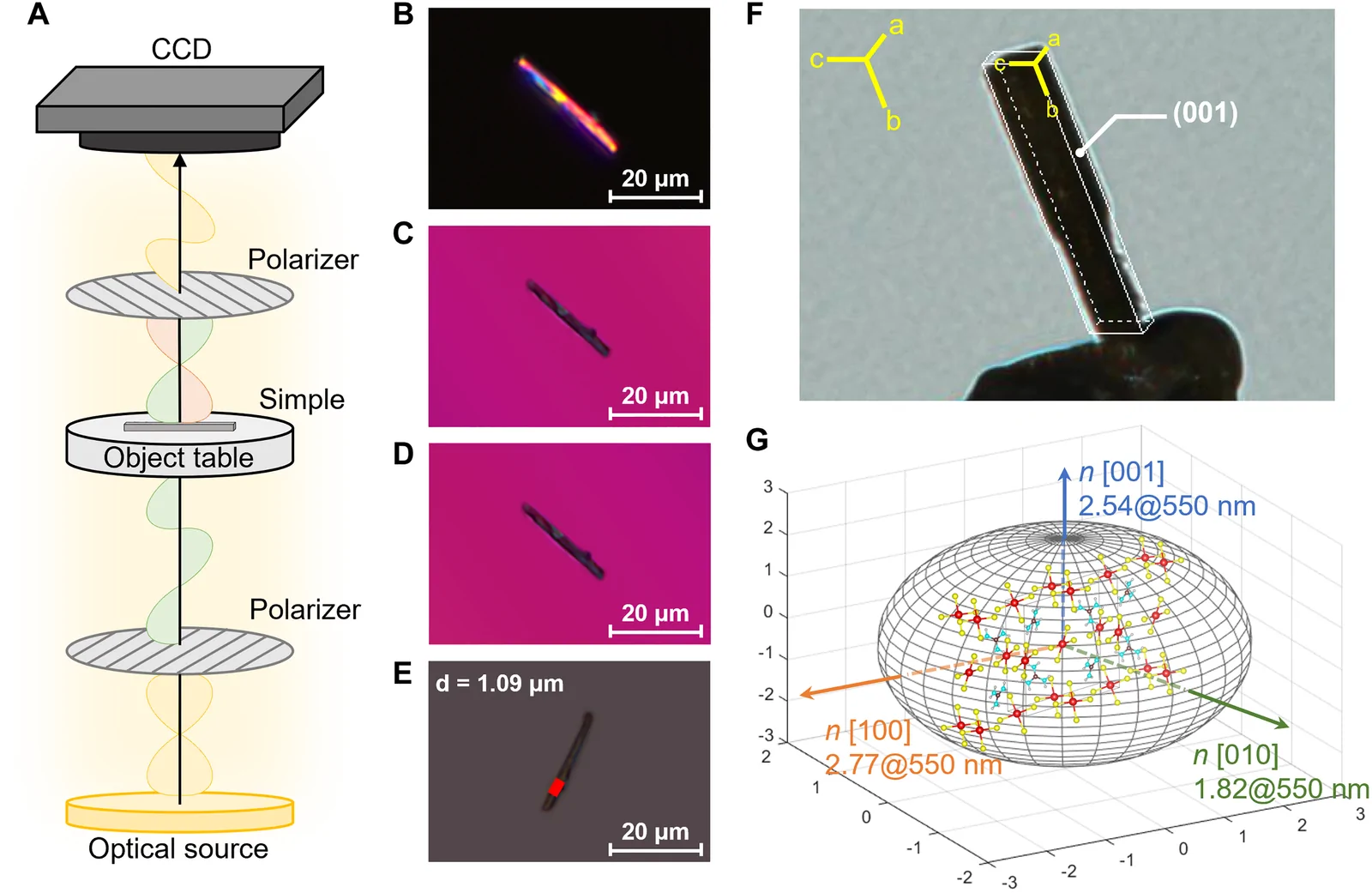
The birefringence measurement of compound [C(NH_2_)_3_][ICl_4_]. (**A**) Schematic diagram of a polarizing microscope. (**B**) Single crystal under a polarizing microscope. (**C**) Crystal completely extinguished under positive compensation of the Berek compensator. (**D**) Crystal completely extinguished under negative compensation of the Berek compensator. (**E**) Crystal thickness. (**F**) (001) crystal plane. (**G**) Refractive index ellipsoid at 550 nm calculated by density functional theory.

In comparison with 141 previously reported compounds featuring tetracoordinate units (e.g., [BX_4_], [SiX_4_], [PX_4_], [SX_4_], [MX_4_], and δ-[MX_4_], where X represents the ligand of the central atom, [MX_4_] represents a metal-centered tetrahedral structure, and δ-[MX_4_] represents a distorted metal-centered tetrahedral coordination structure), the compound in this work exhibits the highest birefringence value (exp. 0.975) reported to date. The detailed birefringence values of all these compounds are listed in table S9. As shown in Fig. 5, the average birefringence values (marked as black hollow circles) of compounds containing tetracoordinate units range from 0.047 (for the [SiX_4_] system) to 0.145 (for the δ-[MX_4_] system).

**Fig. 5.**
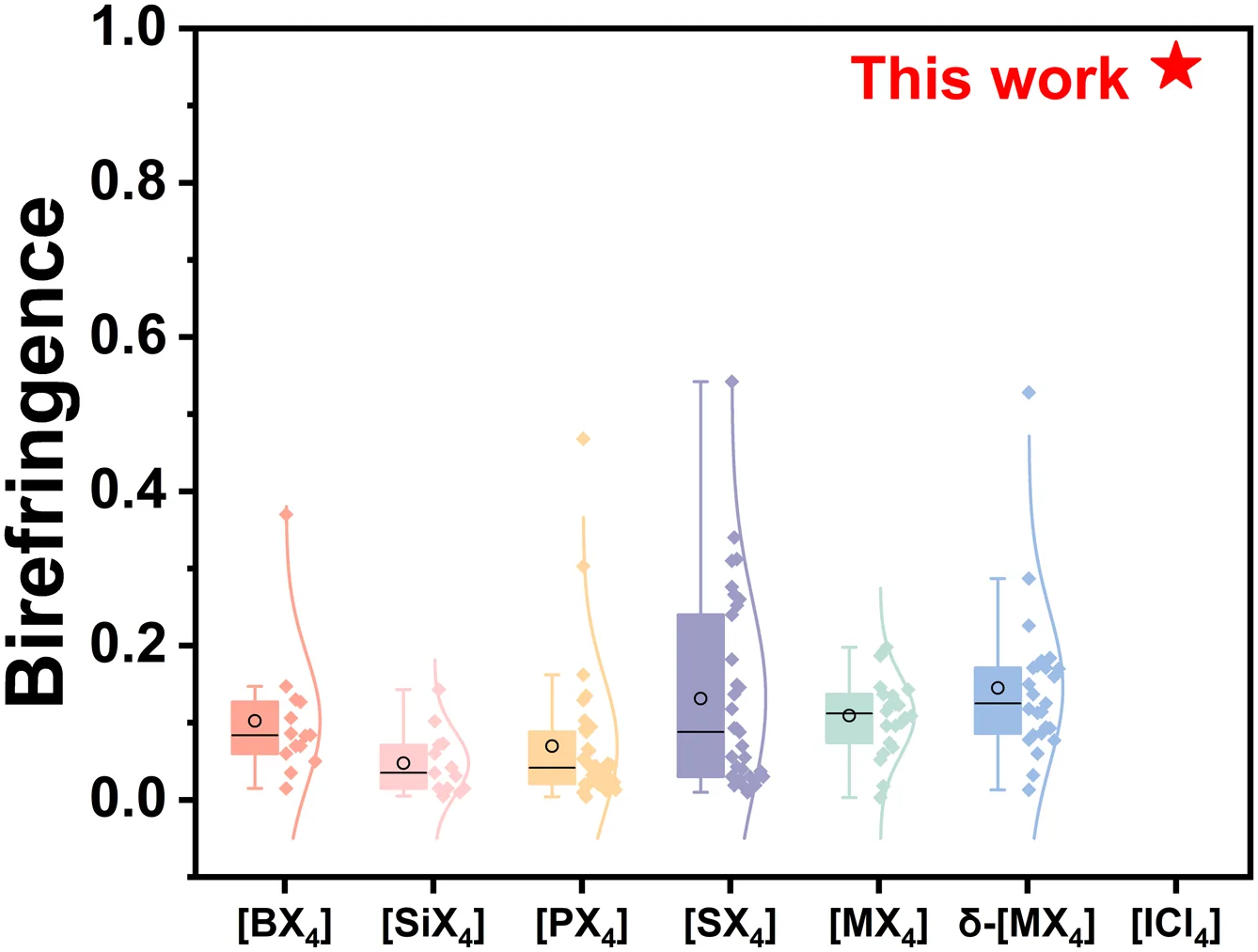
Comparison of the birefringence values between this work and compounds with tetracoordinate units. The hollow black circle denotes the arithmetic mean, the horizontal line inside the box represents the median, the vertical line extending through the box indicates the upper and lower bounds of the data after excluding specified outliers, and the scattered points outside the box show the complete distribution of the raw data.

Furthermore, the median values of birefringence are lower than 0.125 (as shown by the black horizontal line, corresponding to the δ-[MX_4_] system), suggesting that the birefringence values of most compounds with tetracoordinate structural units concentrates at relatively low levels. As mentioned in the introduction, only a handful of known compounds exhibit birefringence values over 0.3. Among these, Hg_4_(Te_2_O_5_)(SO_4_) (*33*) (0.542@546 nm) maintains the highest performance record among all compounds in the current system, while the [C(NH_2_)_3_][ICl_4_] (exp. 0.975@550 nm) reported herein exhibits a 1.75-fold performance enhancement over this long-standing record-holder. In comparison with previously reported non-tetrahedral coordination compounds, the performance of this compound is slightly lower than that of C_3_H_8_N_6_I_6_·3H_2_O (2.8@800 nm) (*15*) but comparable to that of Cs[ICl_2_] (0.994@546 nm) (*39*) and Li_3_(C_9_N_13_)·6H_2_O (1.031@546 nm) (*27*). Thus, compound [C(NH_2_)_3_][ICl_4_] achieves a new milestone in birefringence performance.

To explore the underlying mechanism for the giant birefringence of [C(NH_2_)_3_][ICl_4_], relevant theoretical calculations were conducted herein. The calculated indirect bandgap of [C(NH_2_)_3_][ICl_4_] is 2.53 eV (Fig. 6A), which is in good agreement with the experimental value of 2.34 eV (fig. S4). As shown in Fig. 6B, the total density of states (DOS) and partial DOS of [C(NH_2_)_3_][ICl_4_] preliminarily reveal the origin of birefringence in this compound. Near the Fermi level, the top region of valence band (−5 to 0 eV) is mainly composed of I 5*s*, I 5*p*, Cl 3*p*, C 2*p* and N 2*p* orbitals, while the bottom region of conduction band (0 to 5 eV) is primarily constituted by I 5*p*, Cl 3*p*, C 2*p* and N 2*p* orbitals. This indicates that both [ICl_4_] and [C(NH_2_)_3_] contribute to the birefringence. According to the electron localization function (ELF) plot in fig. S8, the electron density is distributed in both [ICl_4_] and [C(NH_2_)_3_] units, which is consistent with the previous analysis. Specifically, the electron density is mainly concentrated on the delocalized π-electrons of the [C(NH_2_)_3_] unit and the two pairs of lone pair electrons of I atom in the [ICl_4_] unit. Furthermore, frontier molecular orbital analysis of [C(NH_2_)_3_][ICl_4_] (fig. S9) indicates that the HOMO is composed of Cl 3*p* and I 5*p* orbitals, while the LUMO consists of Cl 2*p* orbital and the orbital occupied by two lone-pair electrons on I. For a more intuitive comparison, real-space atomic cutting technique was employed to analyze the birefringence of [C(NH_2_)_3_][ICl_4_]. The results in Fig. 6C reveal that the [ICl_4_] unit exhibits a dominant positive contribution to birefringence, whereas the [C(NH_2_)_3_] unit shows a negative contribution, which is consistent with the above analysis. Although the [C(NH_2_)_3_] cation possesses a planar and π-conjugated structure—features that are typically associated with strong optical anisotropy in guanidinium-based compounds {e.g., [C(NH_2_)_3_][BF_4_] (*40*), see fig. S10}—its contribution in [C(NH_2_)_3_][ICl_4_] is limited. This behavior arises from the exceptionally large polarizability anisotropy of the square-planar [ICl_4_] unit (Δα = 142.1 a.u.), which dominates the optical response.

**Fig. 6.**
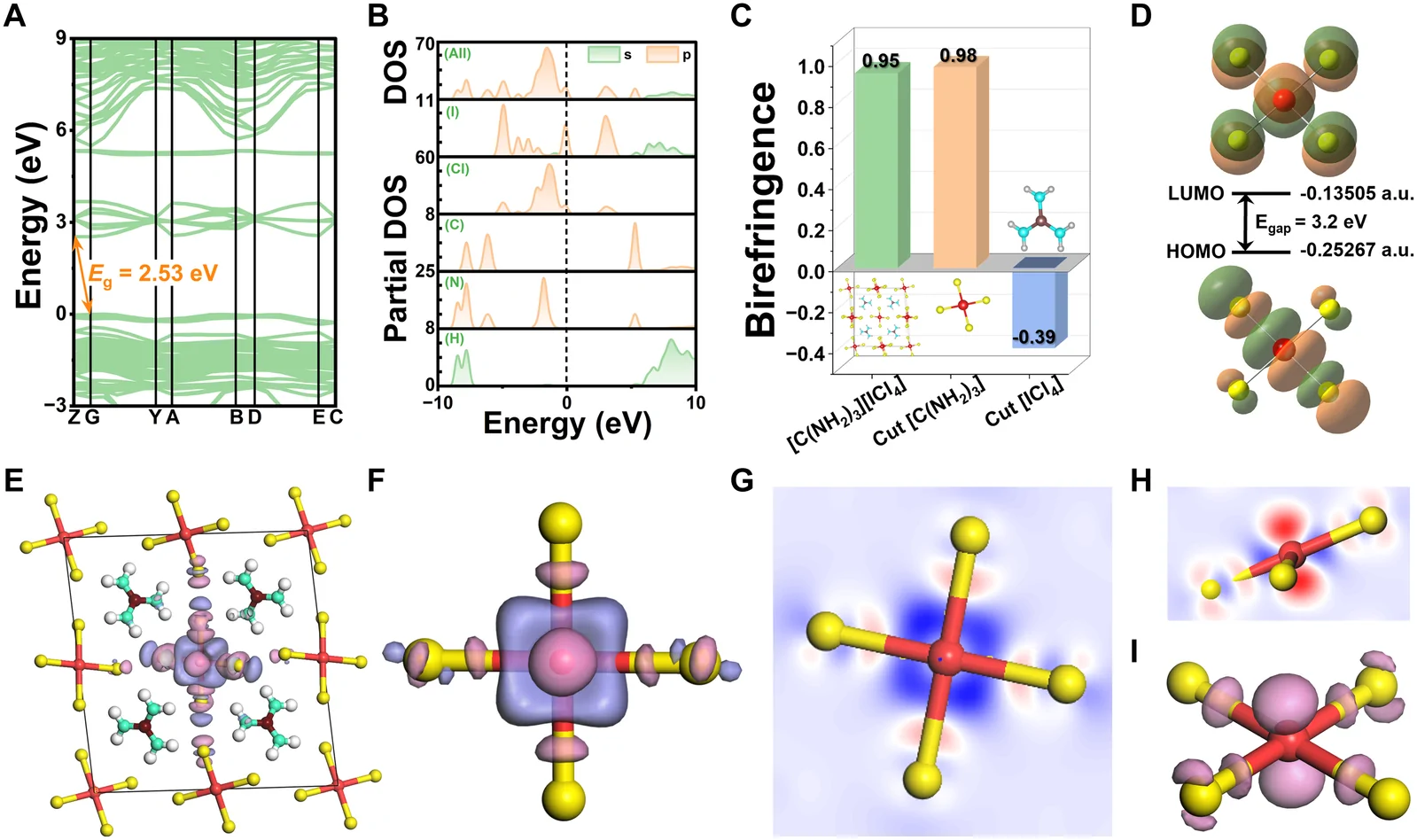
The theoretical calculation of compound [C(NH_2_)_3_][ICl_4_]. (**A**) The calculated electronic band structure of [C(NH_2_)_3_][ICl_4_]. (**B**) The DOS and partial DOS of [C(NH_2_)_3_][ICl_4_]. (**C**) The real-space atomic cutting analysis. (**D**) The HOMO-LUMO of the [ICl_4_] unit. (**E**) Electronic density difference of the [C(NH_2_)_3_][ICl_4_] unit cell. (**F**) Electronic density difference of the [ICl_4_] unit. (**G**) Sliced of electronic density difference for the planar view of [ICl_4_]. (**H**) Slice of electronic density difference for the side view of [ICl_4_]. (**I**) Charge density difference calculations for [ICl_4_].

To investigate the fundamental reason behind the significant contribution of [ICl_4_] to the birefringence of [C(NH_2_)_3_][ICl_4_], we conducted an in-depth theoretical computational analysis of the [ICl_4_] unit. The HOMO-LUMO energy gap of [ICl_4_] was calculated using Gaussian 09 (Fig. 6D), yielding a value of 3.2 eV, which is relatively small. Electronic density difference (EDD) maps clearly elucidate the charge transfer mechanism. As depicted in Fig. 6E, the intramolecular charge transfer within the [ICl_4_] units is stronger than the intermolecular charge transfer between adjacent [ICl_4_] units, whereas the latter is more intense than the interaction between [ICl_4_] and [C(NH_2_)_3_] units. As a result, the strong charge transfer interactions cause significant charge redistribution within the [ICl_4_] units (Fig. 6F). The EDD slices of the [ICl_4_] plane (Fig. 6G) and side perspectives (Fig. 6H) indicate that charges accumulate on the two lone-pair electrons in [ICl_4_] and the σ bonds between I and Cl atoms. Charge density difference calculations (Fig. 6I) further confirm that the polarizability anisotropy of the [ICl_4_] units are largely determined by the two lone-pair electrons of I and the σ-bonds between I and Cl atoms. These findings demonstrate that the two lone-pair electrons on the I atom and σ-bonds together induce polarizability anisotropy in the planar square [ICl_4_] unit, which in turn drives the compound [C(NH_2_)_3_][ICl_4_] to exhibit giant birefringence.

## DISCUSSION

In conclusion, we screened and identified that the [ICl_4_] unit with a square-planar structure exhibits the highest polarizability anisotropy among all tetracoordinate units. An improved synthetic method based on green chemistry principles was used to successfully prepare the crystal [C(NH_2_)_3_][ICl_4_] containing [ICl_4_] unit. The [ICl_4_] anion and [C(NH_2_)_3_] cation construct a zero-dimension packing structure through ionic interactions. The experimental birefringence of compound [C(NH_2_)_3_][ICl_4_] reaches 0.975 at 550 nm, which establishes a record for compounds containing tetra-coordinate structural units. The giant birefringence of this compound arises from the [ICl_4_] unit with square-planar structure, and its excellent polarizability anisotropy arises from the synergistic interaction between two lone-pair electrons and σ-bonds. The unique electronic structure and coordination capability of the [ICl_4_] unit may give it potential value in frontier fields such as catalysis, functional coordination chemistry, and optoelectronic materials. This research presents the [ICl_4_] unit as a new-generation birefringent functional motif, establishing a strategy to modulate polarizability anisotropy through structural configuration design and thereby facilitating the application of high-performance optical materials in optoelectronic devices.

## MATERIALS AND METHODS

### Synthesis and crystal growth

Guanidine hydrochloride (98%) purchased from LEYAN, iodine pentoxide (99%) purchased from RHAWN, phosphoric acid (50% aqueous solution) purchased from RHAWN.

The single crystal of [C(NH_2_)_3_][ICl_4_] was obtained via slow evaporation of its aqueous solution. Dissolve the reagents CH_6_ClN_3_ (0.332 g, 3.5 mmol) and I_2_O_5_ (0.165 g, 0.5 mmol) in 2 mL of 50% phosphoric acid solution. While stirring, purified water was slowly added until a clear solution was obtained. After evaporating at room temperature for 4–5 days, crystals of [C(NH_2_)_3_][ICl_4_] were collected by filtration with a yield of 65.5% (based on I_2_O_5_, see fig. S11). The crystal morphology observed at various magnifications is shown in fig. S12.

### Single crystal structure determination

Single-crystal x-ray diffraction was used to determine the structures of [C(NH_2_)_3_][ICl_4_]. Single-crystal data of [C(NH_2_)_3_][ICl_4_] were collected on a Rigaku XtaLAB at room temperature, using Mo-Kα radiation (λ = 0.71073 Å) with a graphite monochromatic. The crystal structure of this compound was determined by SHELXL (*41*) refinement using OLEX2 (*42*) program. All non-hydrogen atoms are refined using anisotropic thermal parameters. A symmetry check performed with PLATON (*43*) confirmed that no higher symmetric elements were missing. The crystallographic data for [C(NH_2_)_3_][ICl_4_] have been deposited in the Cambridge Crystallographic Data Centre (CCDC) under deposition number 2483461.

### Powder x-ray diffraction

The phase purity of [C(NH_2_)_3_][ICl_4_] was verified by powder x-ray diffraction (PXRD) analysis, conducted on a Miniflex600 diffractometer equipped with Cu Kα radiation (λ = 0.71073 Å). Continuous scans were performed from 5° to 75° in 2θ, with a step width of 0.02° at a speed of 10° min^−1^.

### Thermal analysis

Thermal stability was evaluated using a Rigaku TG-DTA8122 thermal analyzer under dynamic nitrogen flow. Sample was heated from room temperature to 1000°C at a rate of 5°C/min.

### Ultraviolet-visible absorption spectroscopy test

UV-Vis-NIR diffuse reflectance spectra for [C(NH_2_)_3_][ICl_4_] was collected on a PerkinElmer Lamda-950 UV/vis/NIR spectrophotometer. The experiment completed data collection at room temperature, with the wavelength scanning range covering the ultraviolet to near-infrared bands (200–800 nm). High-purity BaSO_4_ powder was used to construct standard samples for reference correction. The recorded data provides details on the diffuse reflectance properties of the samples within the given wavelength range.

### Birefringence measurements

The birefringence of [C(NH_2_)_3_][ICl_4_] was measured using a polarizing microscope with a Berek compensator (Nikon LV100N POL). Birefringence (Δ*n*) was calculated from the optical path difference (*R*) and crystal thickness (*d*) using the formula

$$R=\left(n_{\max}-n_{\min}\right)\times d=\Delta n\times d$$

Here, *R* represents the optical path difference, Δ*n* means the birefringence, and *d* denotes the thickness of the crystal.

### Computational methods

All first-principles calculations were carried out based on density functional theory (DFT) using the CASTEP (*44*, *45*) package. The generalized gradient approximation (GGA) (*46*) with the Perdew–Burke–Ernzerhof (PBE) function was adopted to describe the exchange–correlation interactions. Ultrasoft pseudopotentials were used to model the ion–electron interactions, with a plane-wave cutoff energy of 800 eV to ensure the convergence of the total energy and electronic structure. Given the relatively large unit cell of the studied system, Brillouin zone sampling was performed using a Γ-centered 1 × 4 × 1 *k*-point mesh. Convergence tests with denser k-point grids confirmed negligible changes in the calculated electronic and optical properties, thereby validating the selected sampling scheme. The electronic structures were analyzed by computing the total and partial density of states (DOS), along with the electron localization function (ELF), to evaluate the bonding characteristics. Electron density difference (EDD) maps were further generated to visualize charge redistribution within the crystal. Optical properties, including the frequency-dependent dielectric function and birefringence, were evaluated using linear response theory as implemented in CASTEP. To quantitatively decompose the optical anisotropy into contributions from different structural units, the real-space atom-cutting method was applied.

## Supplementary Materials

### The PDF file includes:

Figs. S1 to S12 Tables S1 to S9 Legend for data file S1 References
