# Supplementary material for: Breaking the tetrahedral constraint of tetracoordination: Square-planar [ICl4] enables unprecedented birefringence in [C(NH2)3][ICl4]
Source: Sci Adv. 2026 Oct 16;12(42):eaef7948. doi: 10.1126/sciadv.aef7948 (PMC13674490; doi:10.1126/sciadv.aef7948)
Supplement: Supplementary file 1 [file sciadv.aef7948_sm.pdf]

Science Advances
AAAS

Supplementary Materials for

Breaking the tetrahedral constraint of tetracoordination: Square-planar $[ICl_4]$ enables unprecedented birefringence in $[C(NH_2)_3][ICl_4]$

Zhiwei Sun *et al.*

Corresponding author: Qingran Ding, dqr@jxnu.edu.cn; Junhua Luo, jhluo@fjirsm.ac.cn;
Sangen Zhao, zhaosangen@quantumsc.cn

Sci. Adv. **12**, eaef7948 (2026)
DOI: 10.1126/sciadv.aef7948

The PDF file includes:

Figs. S1 to S12
Tables S1 to S9
Legend for data file S1
References

Other Supplementary Material for this manuscript includes the following:

Data file S1

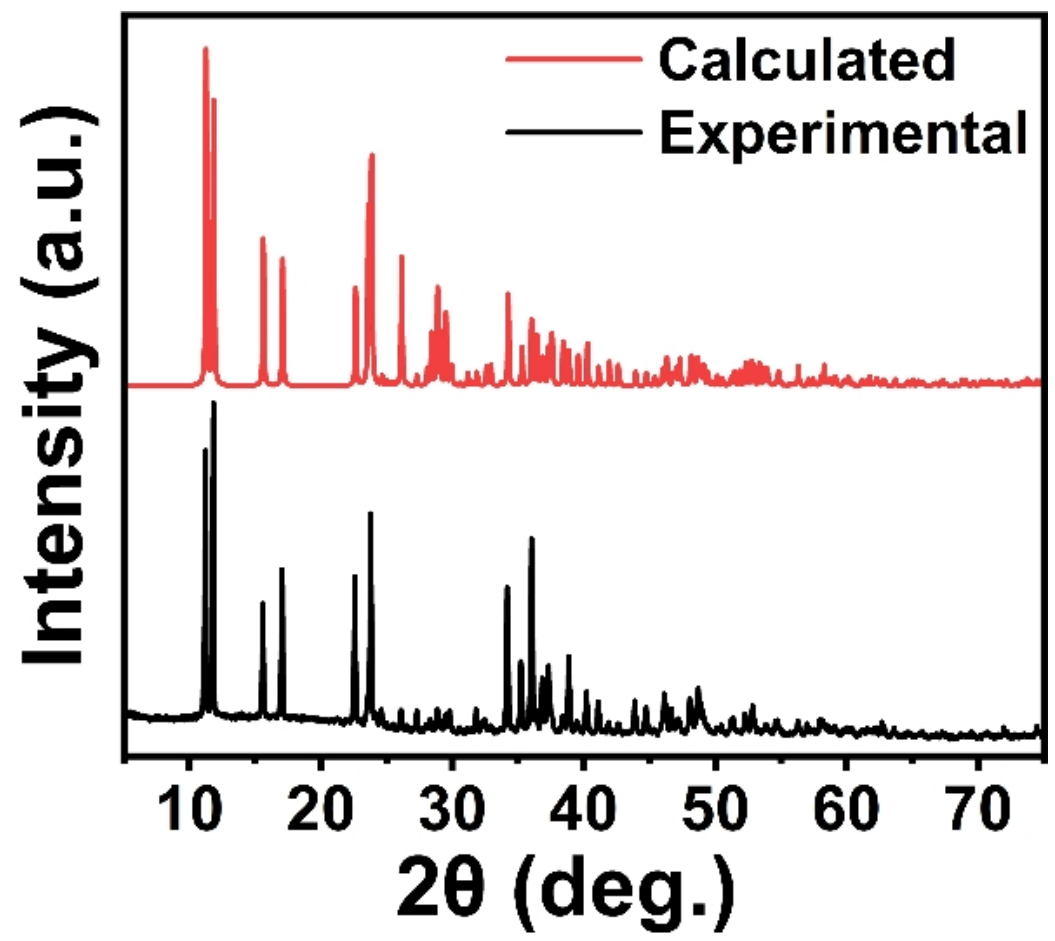

Fig. S1.
Powder XRD patterns of $[C(NH_2)_3][ICl_4]$.

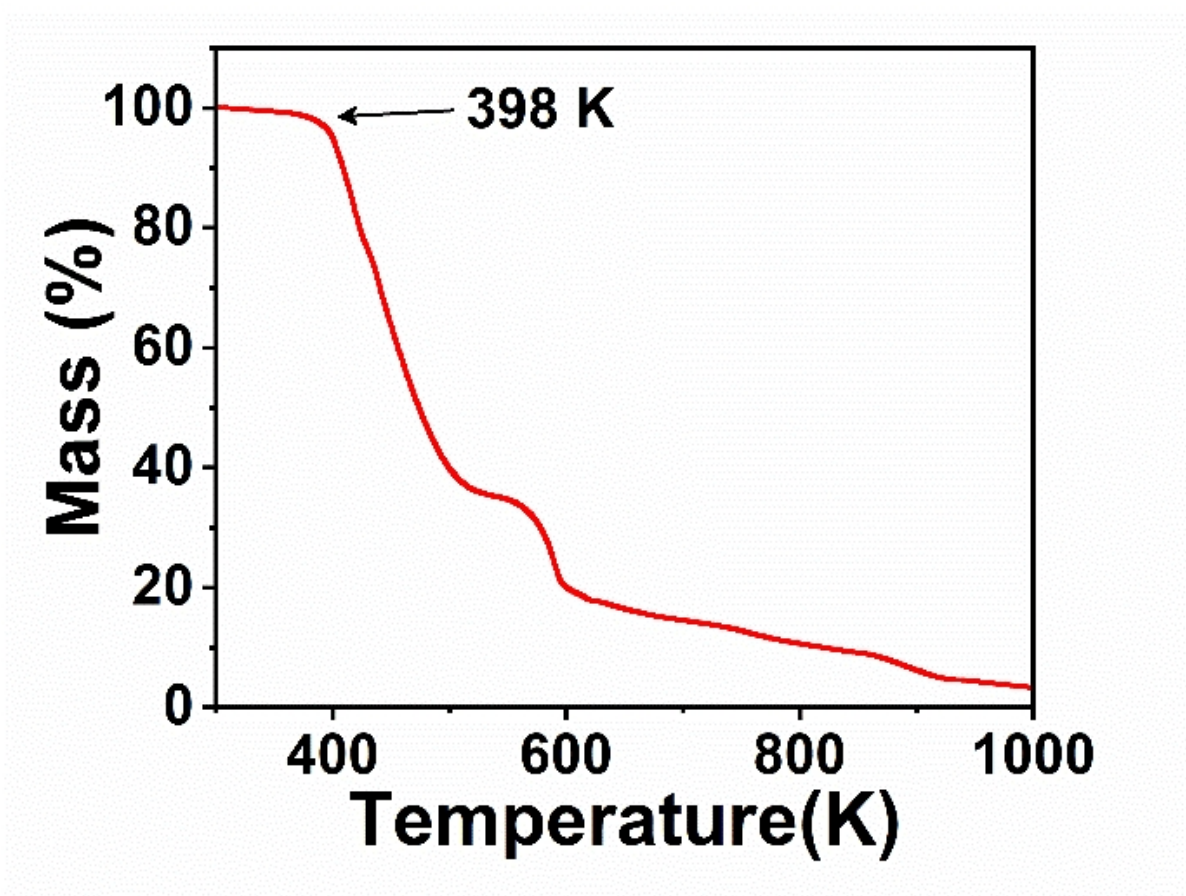

Fig. S2.
The TGA curves of $[C(NH_2)_3][ICl_4]$.

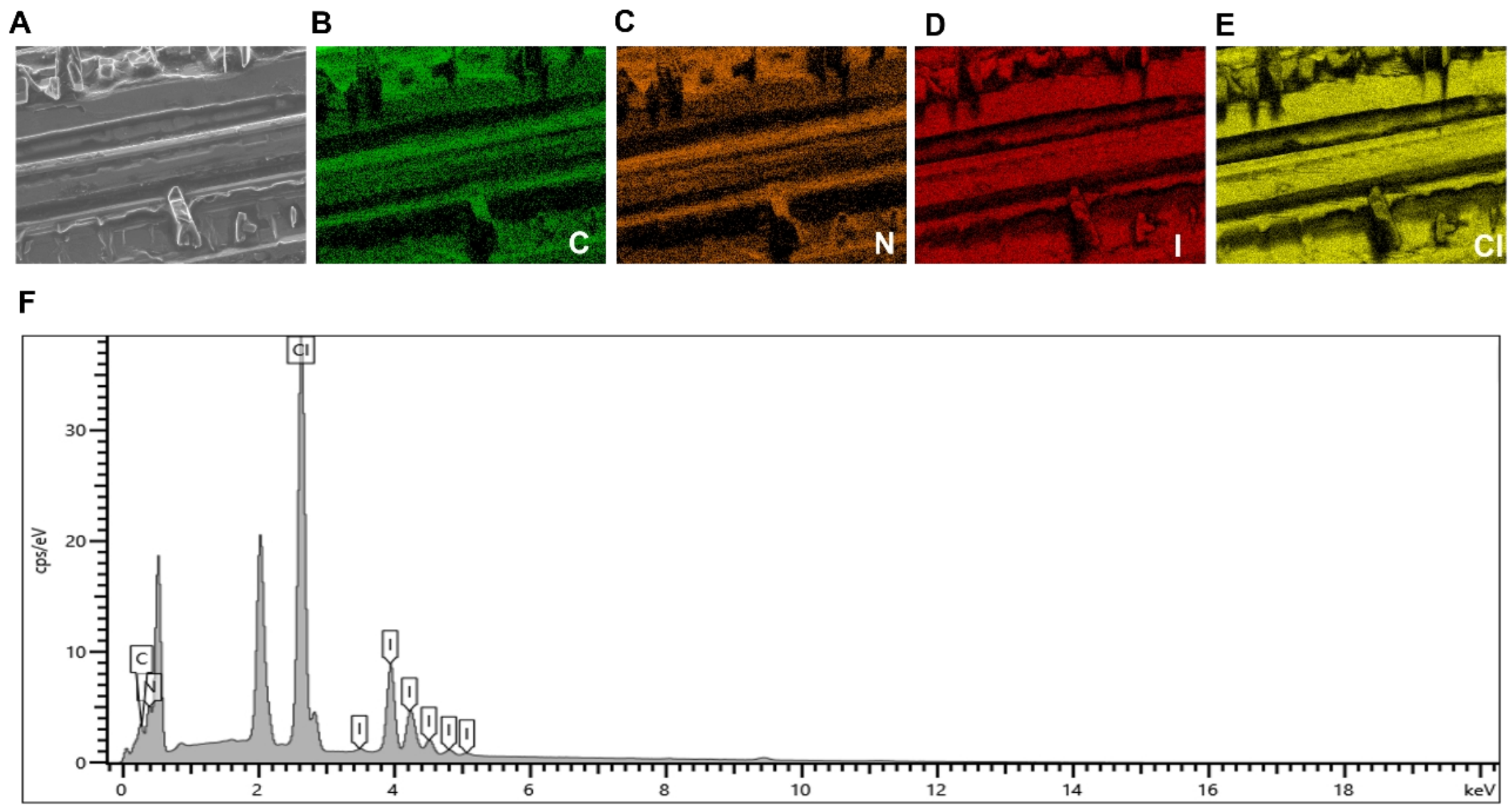

Fig. S3.

The typical energy dispersive X-ray spectroscopy (EDX) elemental mapping images of [$C(NH_2)_3$][ICl_4]. (**A**) The SEM image corresponding to the mapping area. (**B**) C mapping. (**C**) N mapping. (**D**) I mapping. (**E**) Cl mapping. (**F**) EDS images.

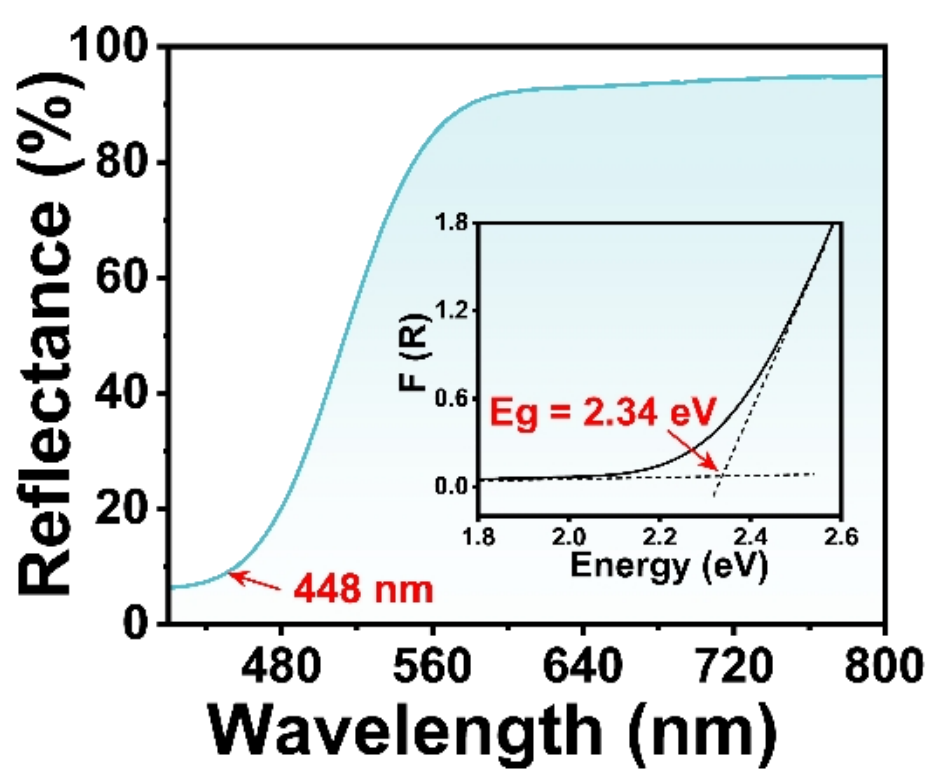

Fig. S4.

The UV-Vis-NIR diffuse reflectance spectra for [$C(NH_2)_3$][ICl_4].

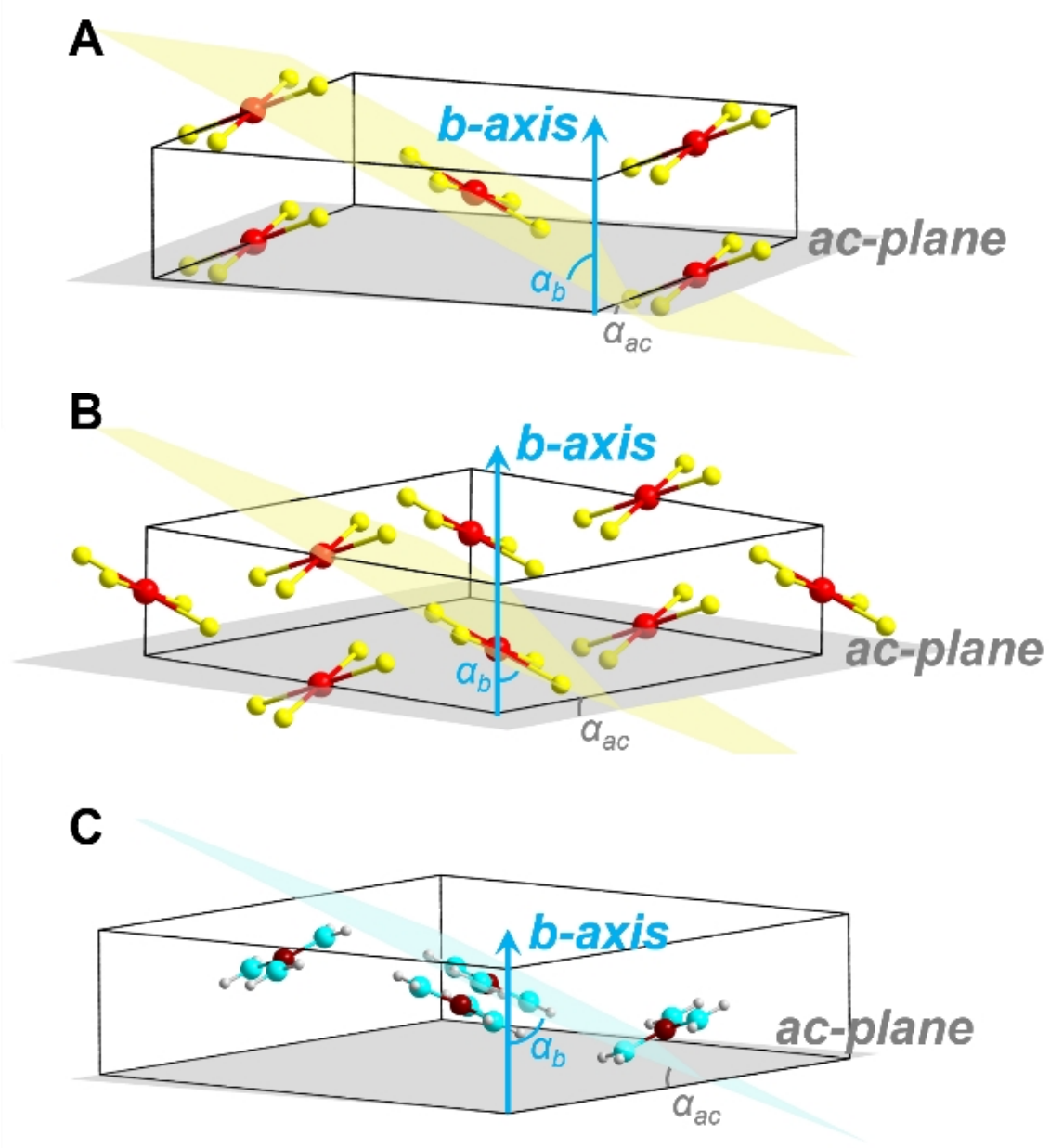

Fig. S5.

Definition of orientation angles of structural units relative to the *b*-axis and *ac*-plane. (**A**) [I1Cl$_4$]. (**B**) [I2Cl$_4$]. (**C**) [C(NH$_2$)$_3$].

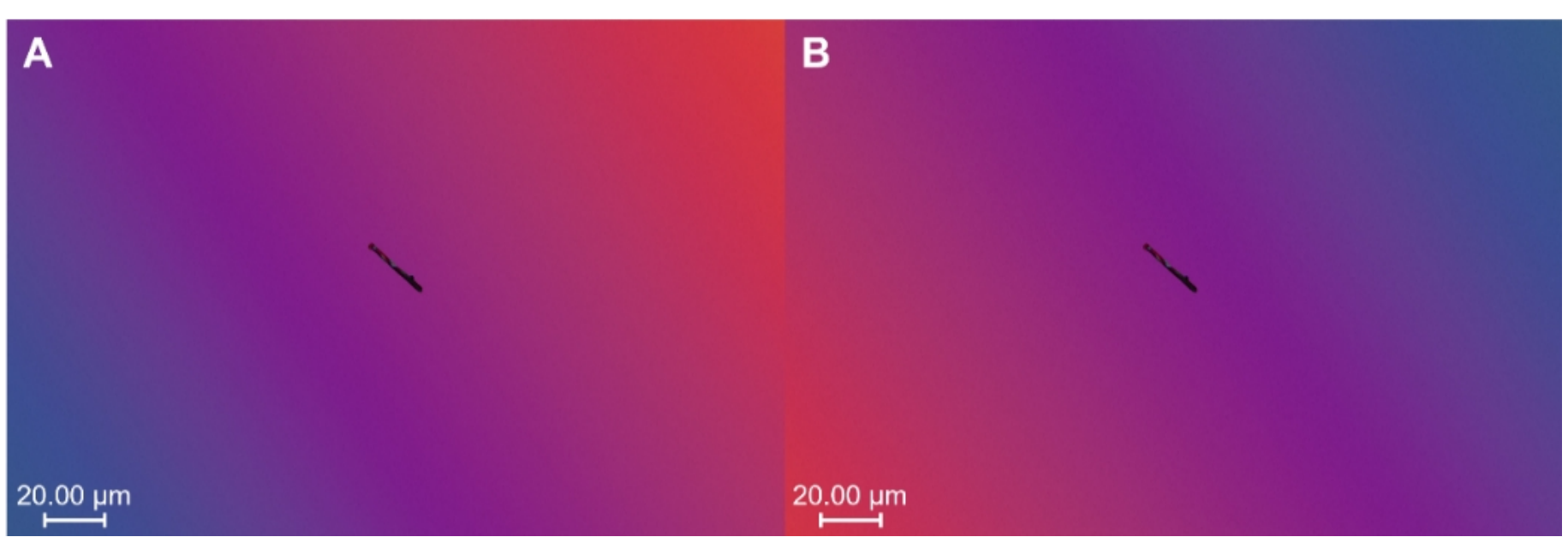

Fig. S6.

The corresponding raw polarized light microscopy images. (A) Crystal completely extinguished under positive compensation of the Berek compensator. **(B)** Crystal completely extinguished under negative compensation of the Berek compensator.

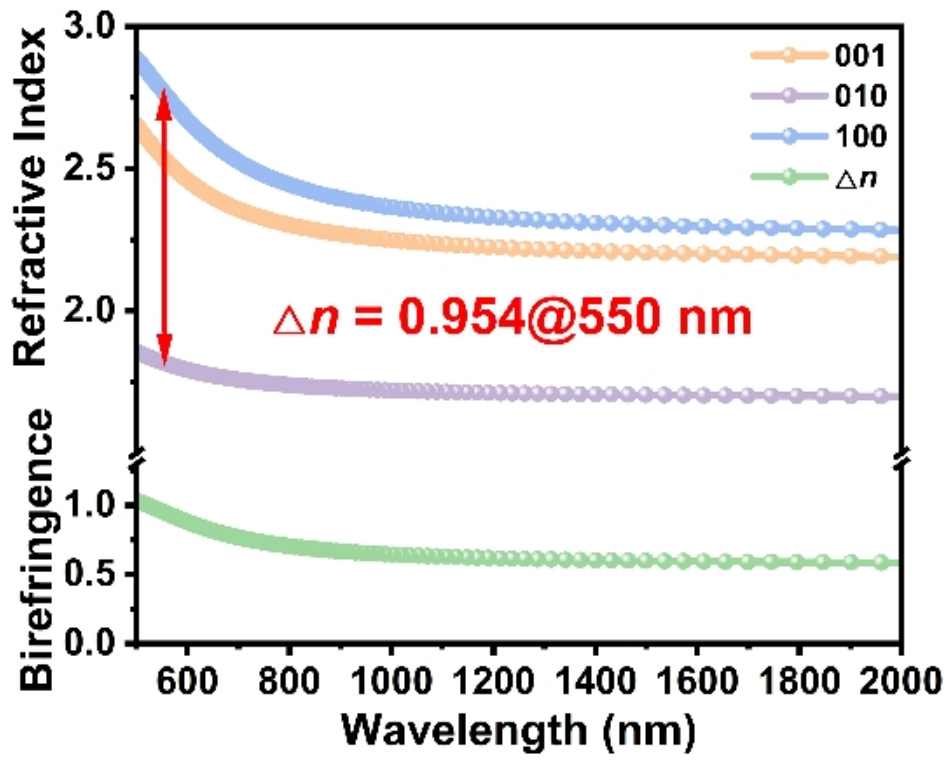

Fig. S7.
The calculated birefringence values of $[C(NH_2)_3][ICl_4]$.

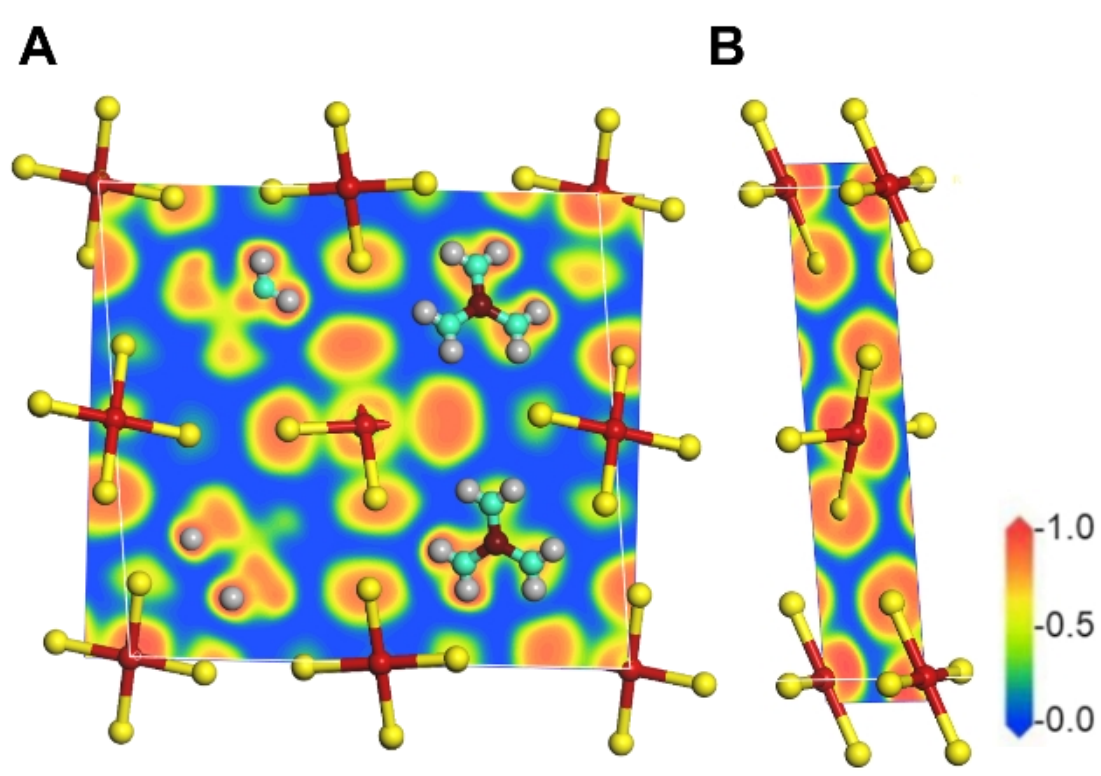

Fig. S8.
The ELF of $[C(NH_2)_3][ICl_4]$. (**A**) *ac*-plane. (**B**) *bc*-plane.

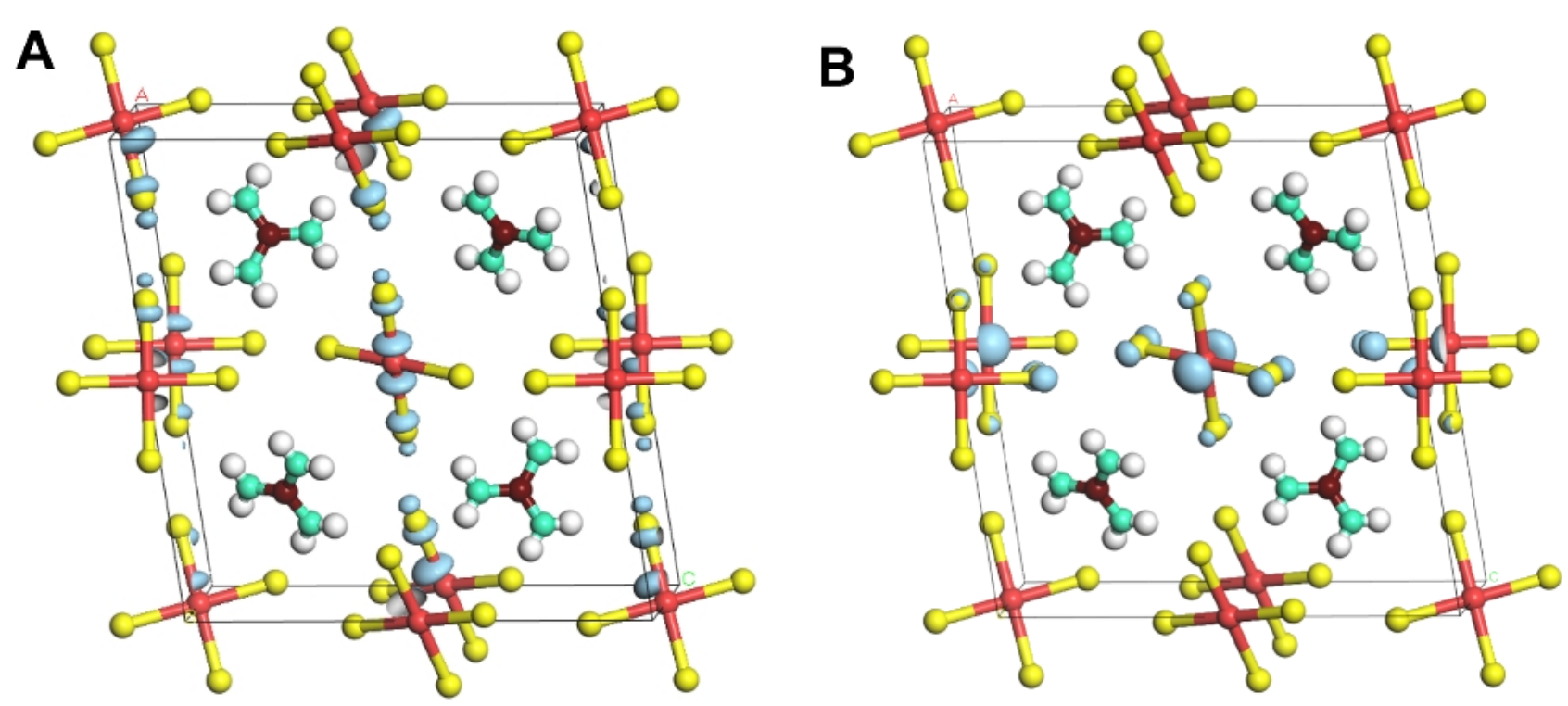

Fig. S9.
Frontier molecular orbital analysis of $[C(NH_2)_3][ICl_4]$. (A) HOMO. **(B)** LUMO.

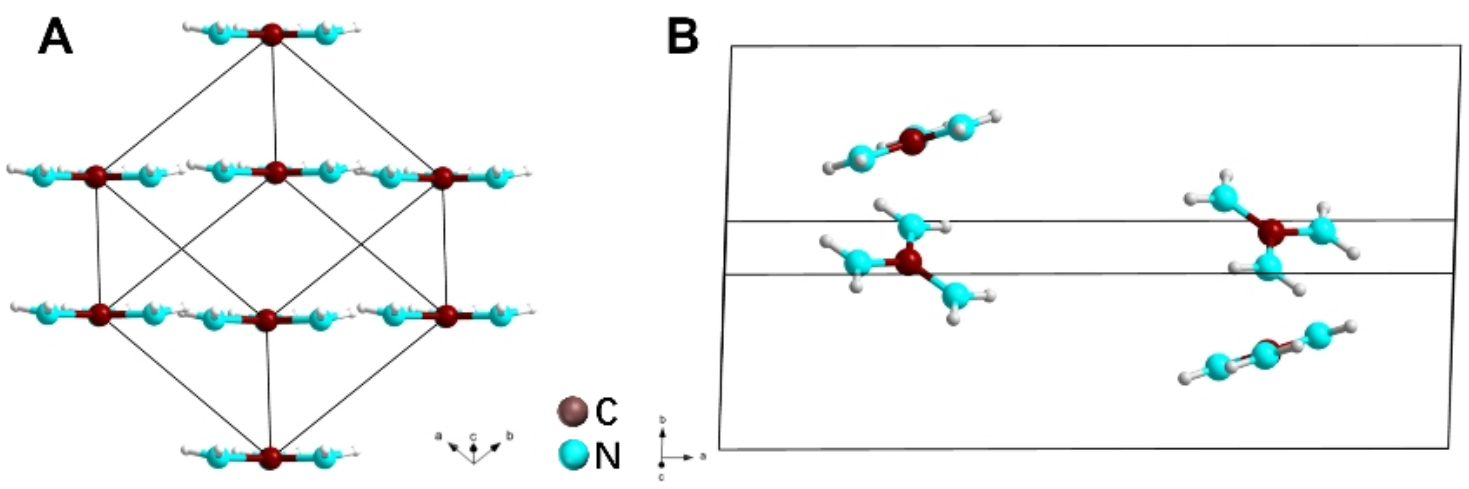

Fig. S10.

The arrangement of $[C(NH_2)_3]$ cations in compound. (**A**) $[C(NH_2)_3]BF_4$. (**B**) $[C(NH_2)_3][ICl_4]$.

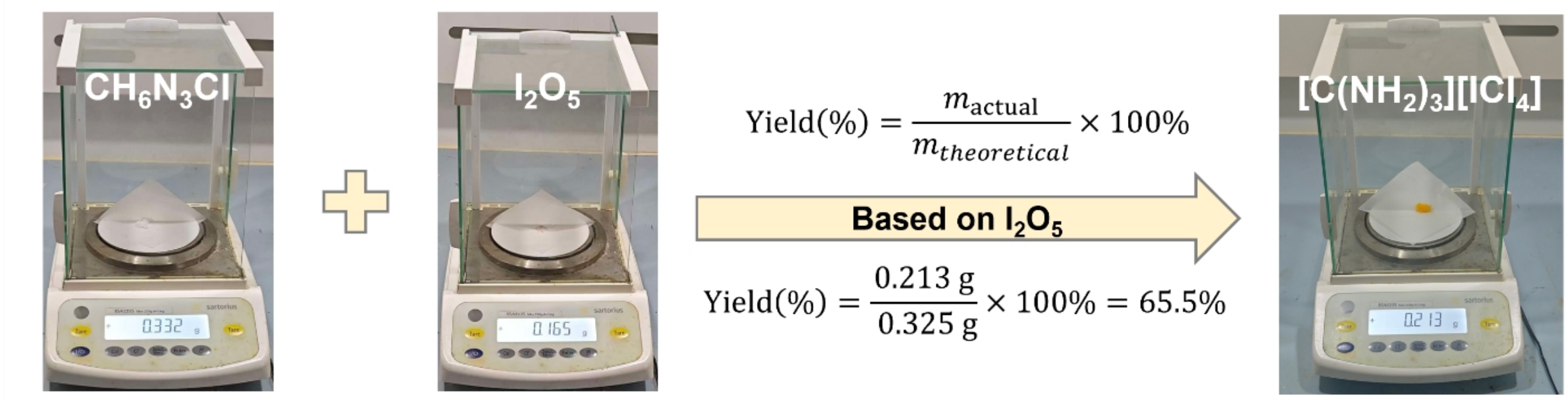

Fig. S11.

Experimental synthesis of $[C(NH_2)_3][ICl_4]$ and calculation of the reaction yield (based on I_2O_5).

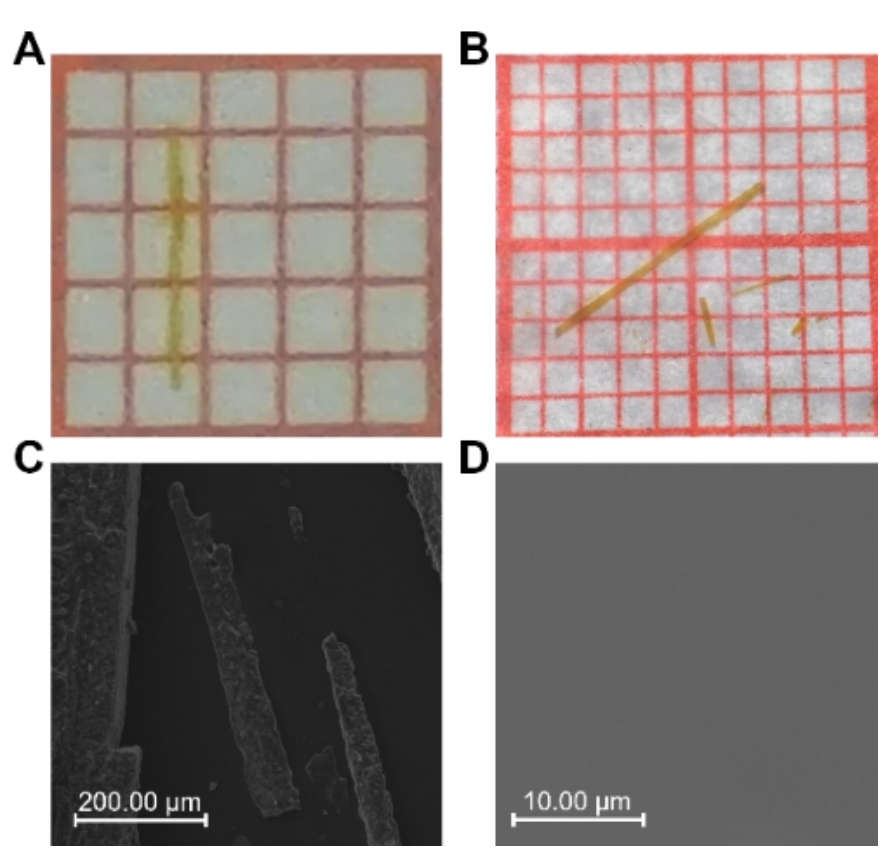

Fig. S12.

Crystal morphology and microscopic characterization. (A) Optical microscope image of a representative single crystal. **(B)** Optical microscope image showing multiple single crystals. **(C)** Low-magnification scanning electron microscopy (SEM) image of the crystals. **(D)** High-magnification SEM image revealing the surface morphology of an individual crystal.

Table S1.

Comparison of microscopic polarizability anisotropy (Δα) between the traditional non-metallic tetrahedral units and [ICl_4] unit.

Unit	Polarizability anisotropy (Δα)	Unit	Polarizability anisotropy (Δα)
[BF_4]	0.2	[PO_2F_2]	6.6
[BO_4]	1.56	[PON_3]	9.2
[BO_3F]	2.1	[PO_2N_2]	10.3
[BOF_3]	2.5	[$POS(OH)_2$]	22.4
[BO_2F_2]	3.4	[$POS(NH_2)_2$]	24.8
[BO_3N]	6.9	[$PO_2S(NH_2)$]	26.7
[SiO_4]	0.0	[PO_3S]	38.2
[SiN_4]	0.0	[POS_3]	39.6
[$SiNO_3$]	4.8	[PO_2S_2]	42.6
[SiN_3O]	5.2	[SO_4]	0.0
[SiN_2O_2]	9.0	[$SO_3(NH_2)$]	2.3
[$SiOS_3$]	47.4	[SO_3F]	4.7
[PO_4]	0.0	[SO_3S]	29.5
[POF_3]	5.3	**[ICl_4]**	**142.1**
[PO_3N]	6.5		

Table S2.

Comparison of microscopic polarizability anisotropy (Δα) between square-planar units and $[ICl_4]$ unit.

	Unit	Polarizability anisotropy (Δα)
Metal-centered	$[Cu(NH_3)_4]$	3.6
	$[Pt(NH_3)_4]$	16.3
	$[Pd(NH_3)_4]$	21.0
	$[PtCl_4]$	55.3
	$[Ni(CN)_4]$	57.6
Non-metallic centered	$[IF_4]$	46.4
	$[BrF_4]$	47.7
	$[XeF_4]$	55.0
	**$[ICl_4]$**	**142.1**

Table S3.
Crystal data and structure refinement for $[C(NH_2)_3][ICl_4]$.

**Compound**	**$[C(NH_2)_3][ICl_4]$**
Formula weight	328.79
Wavelength (Å)	0.71073
Temperature (K)	298
Crystal system, space group	Monoclinic, $P2_1/c$
a (Å)	15.7788(8)
b (Å)	3.9963(2)
c (Å)	15.0029(7)
α (°)	90
β (°)	95.156(5)
γ (°)	90
Volume ($Å^3$)	942.21(8)
Z	4
ρ (g/cm^3)	2.318
Absorption coefficient (mm^{-1})	4.464
F(000)	617.0
Crystal size (mm)	0.6 × 0.6 × 3
2Θ range for data collection (deg.)	7.18 to 58.84
Index ranges	$-19 \le h \le 20$, $-5 \le k \le 5$, $-20 \le l \le 19$
Reflections collected / unique	6409
Independent reflections	2239 [R_{int} = 0.0291, R_{sigma} = 0.0387]
Data/restraints/parameters	2239/0/85
Goodness-of-fit on F^2	0.928
Final R indices [I>2sigma(I)]	R_1 = 0.0361, wR_2 = 0.1238
R indices (all data)	R_1 = 0.0552, wR_2 = 0.1509
Largest diff. peak and hole ($e.A^{-3}$)	0.85/-0.83

$^aR_1(F) = \sum||F_o|-|F_c||/\sum|F_o|$. $wR_2(F_o^2) = [\sum w(F_o^2-F_c^2)^2/\sum w(F_o^2)^2]^{1/2}$

Table S4.

Fractional atomic coordinates (×10^4) and equivalent isotropic displacement parameters (Å^2×10^3) for [C(NH$_2$)$_3$][ICl$_4$]. $U_{(eq)}$ is defined as 1/3 of the trace of the orthogonalized U_{ij} tensor.

Atom	*x*	*y*	*z*	U$_{eq}$ (Å^2)
I1	5000	5000	5000	27.5(2)
I2	10000	10000	5000	27.4(2)
Cl1	5127.3(10)	2699(4)	3469.6(9)	45.1(4)
Cl3	10280.6(11)	12209(4)	6556.3(9)	47.8(4)
Cl2	6534.9(9)	6562(4)	5118.3(11)	50.8(4)
Cl4	8537.3(9)	8190(4)	5294.2(11)	49.0(4)
N3	3185(3)	-944(15)	2855(4)	69.7(17)
N2	1832(4)	-2771(15)	2815(4)	73.6(18)
C1	2520(4)	-2323(19)	2411(4)	45.9(16)
N1	2553(4)	-3233(17)	1588(4)	81(2)

Table S5.
Anisotropic displacement parameters (Å^2 × 10^3) for [C(NH$_2$)$_3$][ICl$_4$].

Atom	U_{11}	U_{22}	U_{33}	U_{12}	U_{13}	U_{23}
I1	27.5(3)	29.5(4)	25.9(3)	-5.86(16)	5.1(2)	0.18(16)
I2	25.2(3)	28.3(4)	28.6(3)	-0.72(15)	2.3(2)	3.70(17)
Cl1	51.5(9)	54.9(9)	30.1(7)	-9.0(7)	9.7(6)	-9.1(6)
Cl3	60.4(10)	50.9(9)	31.3(7)	1.1(7)	-0.3(6)	-5.5(6)
Cl2	29.5(7)	66.1(11)	57.5(10)	-14.4(7)	8.1(6)	-12.2(8)
Cl4	31.0(7)	57.8(10)	59.3(10)	-9.2(6)	9.5(6)	6.4(8)
N3	49(4)	104(5)	55(4)	-17(4)	-4(3)	-28(3)
N2	52(4)	112(5)	60(4)	-9(4)	24(3)	-7(4)
C1	44(4)	57(4)	37(3)	-2(3)	7(3)	-2(3)
N1	71(4)	131(6)	44(3)	-35(4)	16(3)	-24(4)

The anisotropic displacement factor exponent takes the form: $-2\pi^2[h^2a^{*2}U_{11}+2hka^*b^*U_{12}+\ldots]$.

Table S6.
Selected bond lengths (Å) for $[C(NH_2)_3][ICl_4]$.

Atom-Atom	Length/Å	Atom-Atom	Length/Å
I1-Cl1	2.4982(13)	I2-Cl4	2.4956(14)
I1-Cl1[1]	2.4982(13)	I2-Cl4[2]	2.4956(14)
I1-Cl2	2.4918(14)	C1-N1	1.292(7)
I1-Cl2[1]	2.4918(14)	C1-N2	1.303(6)
I2-Cl3	2.4983(13)	C1-N3	1.312(7)
I2-Cl3[2]	2.4983(13)		

[1]1-X,1-Y,1-Z; [2]2-X,2-Y,1-Z

Table S7.
Selected bond angles (°) for $[C(NH_2)_3][ICl_4]$.

Atom-Atom-Atom	Angle/°	Atom-Atom-Atom	Angle/°
$Cl1^{1}$-I1-Cl1	180.0	Cl4-I2-$Cl3^{2}$	88.50(5)
$Cl2^{1}$-I1-Cl1	90.01(5)	$Cl4^{2}$-I2-$Cl3^{2}$	91.50(5)
$Cl2^{1}$-I1-$Cl1^{1}$	89.99(5)	$Cl4^{2}$-I2-Cl3	88.50(5)
Cl2-I1-$Cl1^{1}$	90.01(5)	Cl4-I2-$Cl4^{2}$	180.0
Cl2-I1-Cl1	89.99(5)	N2-C1-N3	118.9(6)
$Cl2^{1}$-I1-Cl2	180.0	N1-C1-N3	120.2(6)
$Cl3^{2}$-I2-Cl3	180.0	N1-C1-N2	120.9(6)
Cl4-I2-Cl3	91.50(5)		

^{1}1-X,1-Y,1-Z; ^{2}2-X,2-Y,1-Z

Table S8.
The angles between the units of $[C(NH_2)_3][ICl_4]$ and different reference systems.

Unit	Reference system	Angle/°
$[I1Cl_4]$	*a*-axis	11.7
	b-axis	63.6
	c-axis	22.1
	ac-plane	26.4
$[I2Cl_4]$	*a*-axis	19.4
	b-axis	63.2
	c-axis	15.9
	ac-plane	26.8
$[C(NH_2)_3]$	*a*-axis	17.5
	b-axis	64.6
	c-axis	16.0
	ac-plane	25.4

Table S9.

Comparison of the birefringence values of this work with those compounds containing tetrahedral units.

	Compound	Units	Birefringence
		**[BX_4]**	
1	$KC_3H_5BF_3$(*47*)	[$BF_3(C_3H_5)$]	0.015@546 nm (exp.)
2	$CaB_4O_5F_4$-III(*48*)	[BO_2F_2]	0.035@1064 nm (cal.)
3	$Li_2B_3O_4F_3$(*49*)	[BO_2F_2]	0.050@1064 nm (cal.)
4	LiB_6O_9F(*49*)	[BO_3F]	0.060@1064 nm (cal.)
5	$Li_2B_6O_9F_2$(49)	[BO_2F_2]	0.070@1064 nm (cal.)
6	$CaB_4O_5F_4$-IV(*48*)	[BO_2F_2]	0.070@1064 nm (cal.)
7	$CaB_6O_8F_4$-II(*48*)	[BO_2F_2]	0.083@1064 nm (cal.)
8	$CaB_4O_5F_4$-I(*48*)	[BO_2F_2]	0.084@1064 nm (cal.)
9	$CaB_4O_5F_4$-II(*48*)	[BO_2F_2]	0.086@1064 nm (cal.)
10	$CaB_6O_8F_4$-I(*48*)	[BO_2F_2]	0.106@1064 nm (cal.)
11	$CON_3H_6BF_4$(*50*)	[BF_4]	0.127@546 nm (exp.)
12	$C_5H_5BF_3N$(*47*)	[$BF_3(C_5NH_5)$]	0.130@546 nm (exp.)
13	$(C_9H_7BrN)BF_4$(*51*)	[BF_4]	0.147@550 nm (exp.)
14	$(C_3N_6H_7)BF_4\cdot H_2O$(*24*)	[BF_4]	0.370@546 nm (exp.)
		**[SiX_4]**	
15	$Y_2Si_3O_3N_4$(*52-54*)	[SiO_2N_2] and [SiN_4]	0.005@1064 nm (cal.)
16	$Ba_3Si_6O_9N_4$(*52, 55*)	[SiO_2N_2]	0.010@1064 nm (cal.)
17	$Ba_3Si_6O_{12}N_2$(*55*)	[SiO_3N]	0.015@1064 nm (cal.)
18	$SrSiAl_2N_2O_3$(*52*)	[$SiON_3$]	0.015@1064 nm (cal.)
19	$BaSi_6ON_8$(*52 56*)	[$SiON_3$]	0.015@1064 nm (cal.)
20	$BaSi_2O_2N_2$(*57*)	[$SiON_3$]	0.031@1064 nm (cal.)
21	$CaSi_2O_2N_2$(*52, 58*)	[$SiON_3$]	0.035@1064 nm (cal.)
22	$BaAl_2Si_3O_4N_4$(*55*)	[$SiON_3$]	0.042@1064 nm (cal.)
23	LiSiON(*52*)	[$SiON_3$]	0.060@1064 nm (cal.)
24	$Na_6Si_3OS_8$(*59*)	[$SiOS_3$] and [SiS_4]	0.071@1064 nm (cal.)
25	$BaAlSi_4O_3N_5$(*55, 60*)	[$SiON_3$] and [SiN_4]	0.073@1064 nm (cal.)
26	Si_2ON_2(*55, 61*)	[$SiON_3$]	0.102@1064 nm (cal.)
27	$YSiO_2N$(*62*)	[SiO_2N_2]	0.143@1064 nm (cal.)
		**[PX_4]**	
28	$Ba_3P_6O_6N_8$(*63*)	[PON_3]	0.004@1064 nm (cal.)
29	$BaPO_3F$(*64*)	[PO_3F]	0.010@1064 nm (cal.)
30	$Sr_3P_6O_6N_8$(*65*)	[PON_3]	0.012@1064 nm (cal.)
31	Ba_2PO_3N(*66*)	[PO_3N]	0.013@1064 nm (cal.)
32	Cs_2PO_3F(*64*)	[PO_3F]	0.018@1064 nm (cal.)
33	Rb_2PO_3F(*64*)	[PO_3F]	0.020@1064 nm (cal.)
34	$Na_3POS_3\cdot 11H_2O$(*67*)	[POS_3]	0.020@1064 nm (cal.)
35	K_2PO_3F(*64*)	[PO_3F]	0.021@1064 nm (cal.)

36	$NaK_3(PO_3F)_2$(*64*)	$[PO_3F]$	0.021@1064 nm (cal.)
37	$K_2P_2O_5F_2$(*64*)	$[PO_3F]$	0.023@1064 nm (cal.)
38	$(NH_4)_2PO_3F$(*64*)	$[PO_3F]$	0.027@1064 nm (cal.)
39	Na_2PO_3F(*64*)	$[PO_3F]$	0.028@1064 nm (cal.)
40	$Na_3PO_2S_2\cdot 11H_2O$(*67*)	$[PO_2S_2]$	0.030@1064 nm (cal.)
41	Sr_2PO_3N(*68*)	$[PO_3N]$	0.032@1064 nm (cal.)
42	$NaNH_4PO_3F\cdot H_2O$(*69*)	$[PO_3F]$	0.035@532 nm (cal.)
43	$(NH_4)_2PO_3F$(*69*)	$[PO_3F]$	0.035@532 nm (cal.)
44	$[C(NH_2)_3]_2PO_3F$(*69*)	$[PO_3F]$	0.039@532 nm (cal.)
45	$Na_3PO_2S_2$(*70*)	$[PO_2S_2]$	0.044@1064 nm (cal.)
46	Ca_2PO_3N(*68*)	$[PO_3N]$	0.044@1064 nm (cal.)
47	$CsPO_2F_2$(*64*)	$[PO_2F_2]$	0.045@1064 nm (cal.)
48	$NH_4PO_2F_2$(*64*)	$[PO_2F_2]$	0.046@1064 nm (cal.)
49	KPO_2F_2(*64*)	$[PO_2F_2]$	0.047@1064 nm (cal.)
50	$NaNH_4PO_3F\cdot H_2O$(*71*)	$[PO_3F]$	0.053@589.3 nm (cal.)
51	$(NH_4)POS(NH_2)_2$(*72*)	$[POS(NH_2)_2]$	0.064@1064 nm (cal.)
52	Li_2PO_2N(*73, 74*)	$[PO_2N_2]$	0.066@1064 nm (cal.)
53	Na_3PO_3S(*70, 75*)	$[PO_3S]$	0.088@1064 nm (cal.)
54	$Li_{14}(PON_3)_2O$(*76*)	$[PON_3]$	0.092@1064 nm (cal.)
55	$(NH_4)_2PO_2S(NH_2)$(*77*)	$[PO_2S(NH_2)]$	0.095@1064 nm (cal.)
56	Sn_2PO_4F(*78*)	$[PO_4]$	0.103@546 nm (cal.)
57	KH_2PO_3S(*70, 79*)	$[POS](OH)_2]$	0.129@1064 nm (cal.)
58	Na_3POS_3(*70*)	$[POS_3]$	0.135@1064 nm (cal.)
59	Sn_2PO_4Cl(*78*)	$[PO_4]$	0.162@546 nm (cal.)
60	Sn_2PO_4Br(*80*)	$[PO_4]$	0.303@1064 nm (cal.)
61	Sn_2PO_4I(*80*)	$[PO_4]$	0.468@1064 nm (cal.)
		**$[SX_4]$**	
62	$Na_{10}Cd(NO_3)_4(SO_3S)_4$(*81*)	$[SO_3S]$	0.010@1064 nm (cal.)
63	$In_2(TeO_3)_2(SO_4)(H_2O)$(*82*)	$[SO_4]$	0.016@532 nm (cal.)
64	$Na_{10}Zn(NO_3)_4(SO_3S)_4$(*83*)	$[SO_3S]$	0.017@550 nm (cal.)
65	KSO_3F(*84*)	$[SO_3F]$	0.019@546 nm (cal.)
66	$(NH_4)_2S_2O_3$(*85*)	$[SO_3S]$	0.019@546 nm (cal.)
67	$Te_2O_3(SO_4)$(*86*)	$[SO_4]$	0.026@546 nm (cal.)
68	$CsSO_3F$(*85, 87*)	$[SO_3F]$	0.029@1064 nm (cal.)
69	$Ba(NH_2SO_3)_2$(*88*)	$[SO_3(NH_2)]$	0.030@1064 nm (cal.)
70	$Sr(NH_2SO_3)_2$(*88*)	$[SO_3(NH_2)]$	0.030@1064 nm (cal.)
71	$Te(OH)_3(SO_4)\cdot H_3O$(*86*)	$[SO_4]$	0.030@546 nm (cal.)
72	$Cd(NH_2SO_3)_2$(*89*)	$[SO_3(NH_2)]$	0.037@546 nm (cal.)
73	$Ba(CH_3SO_3)_2$(*90*)	$[SO_3(NH_2)]$	0.040@589.3 nm (cal.)
74	$(NH_4)SO_3F$(*85, 91*)	$[SO_3F]$	0.043@1064 nm (cal.)
75	$LiSO_3F$(*85, 92*)	$[SO_3F]$	0.055@1064 nm (cal.)
76	$In_2(TeO_3)(SO_4)(OH)_2(H_2O)$(*93*)	$[SO_4]$	0.056@1064 nm (cal.)
77	$SO_2(NH_2)_2$(*94*)	$[SO_2(NH_2)_2]$	0.070@589.3 nm (exp.)
78	$In_3(TeO_3)(SO_4)F_3(H_2O)$(*93*)	$[SO_4]$	0.088@1064 nm (cal.)
79	$Y_3(TeO_3)_2(SO_4)_2(OH)(H_2O)$(*95*)	$[SO_4]$	0.092@532 nm (cal.)

80	$Sb_2(TeO_3)(SO_4)_2$-$P2_1/c$(II)(*96*)	[SO_4]	0.093@546 nm (cal.)
81	$Sb_2(TeO_3)(SO_4)_2$-P-1(I)(*96*)	[SO_4]	0.118@546 nm (cal.)
82	$Ti(TeO_3)(SO_4)$(*97*)	[SO_4]	0.137@1064 nm (cal.)
83	$Hg_2(TeO_3)(SO_4)$(*33*)	[SO_4]	0.146@546 nm (cal.)
84	$Y_2(Te_4O_{10})(SO_4)$(*95*)	[SO_4]	0.149@532 nm (cal.)
85	$Hg_3(Te_3O_8)(SO_4)$(*33*)	[SO_4]	0.182@546 nm (cal.)
86	$Rb(3\text{-}C_5H_4NSO_3)$(*32*)	[$SO_3(3\text{-}C_5H_4N)$]	0.240@546 nm (cal.)
87	$(NH_4)(3\text{-}C_5H_4NSO_3)$(*32*)	[$SO_3(3\text{-}C_5H_4N)$]	0.252@546 nm (cal.)
88	$Li(3\text{-}C_5H_4NSO_3)$(*32*)	[$SO_3(3\text{-}C_5H_4N)$]	0.260@546 nm (cal.)
89	$Cs(3\text{-}C_5H_4NSO_3)$(*32*)	[$SO_3(3\text{-}C_5H_4N)$]	0.266@546 nm (cal.)
90	$Ag(3\text{-}C_5H_4NSO_3)$(*32*)	[$SO_3(3\text{-}C_5H_4N)$]	0.276@546 nm (cal.)
91	$(C_3N_6H_7)SO_3CH_3\cdot H_2O$(*24*)	[SO_3CH_3]	0.310@546 nm (exp.)
92	$K(3\text{-}C_5H_4NSO_3)$(*32*)	[$SO_3(3\text{-}C_5H_4N)$]	0.312@546 nm (cal.)
93	$C_3N_6H_7SO_3NH_2$(*25*)	[SO_3NH_2]	0.340@546 nm (exp.)
94	$Hg_4(Te_2O_5)(SO_4)$(*33*)	[SO_4]	0.542@546 nm (cal.)
		**[MX_4]**	
95	$K_3GeO_3(NH_2)$(*98*)	[$GeO_3(NH_2)$]	0.003@1064 nm (cal.)
96	KGeON(*99*)	[$GeON_3$]	0.018@1064 nm (cal.)
97	$Na_3AsO_2S_2\cdot 11H_2O$(*100*)	[AsO_2S_2]	0.052@1064 nm (cal.)
98	CdI_3(HDABCO)(*101*)	[CdI_3N]	0.060@546 nm (cal.)
99	$Na_3AsO_3S\cdot 7H_2O$(*102*)	[AsO_3S]	0.068@1064 nm (cal.)
100	$H_{11}C_4N_2CdI_3$(*103*)	[$CdI_3(H_{11}C_4N_2)$]	0.074@1064 nm (cal.)
101	Ge_2ON_2(*104, 105*)	[$GeON_3$]	0.096@1064 nm (cal.)
102	$Na_2GeS_2(OH)_2(H_2O)_5$(*106*)	[$GeS_2(OH)_2$]	0.096@1064 nm (cal.)
103	$K_3(H_2O)GeS_3(OH)$(*107*)	[$GeS_3(OH)$]	0.100@1064 nm (cal.)
104	$Sr_2GeGa_2OS_6$(*108*)	[$GeOS_3$]	0.106@1064 nm (cal.)
105	$Ba_2ZnGe_2O_8S_6$(*109*)	[$GeOS_3$]	0.109@1064 nm (cal.)
106	$Sr_5Ga_8O_3S_{14}$(*110*)	[$GaOS_3$] and [GaS_4]	0.115@1064 nm (cal.)
107	$La_2ZnGa_2OS_6$(*111*)	[$GaOS_3$]	0.122@1064 nm (cal.)
108	$Sr_2ZnGe_2OS_6$(*108*)	[$GeOS_3$]	0.123@1064 nm (cal.)
109	$BaGeOS_2$(*112*)	[GeO_2S_2]	0.124@1064 nm (cal.)
110	$Ca_2GeGa_2OS_6$(*108*)	[$GeOS_3$]	0.135@1064 nm (cal.)
111	$SrGeOS_2$(*112*)	[GeO_2S_2]	0.137@1064 nm (cal.)
112	$Sr_2CdGe_2OS_6$(*108*)	[$GeOS_3$]	0.143@1064 nm (cal.)
113	$Sr_2ZnGe_2O_8S_6$(*113*)	[$GeOS_3$]	0.146@1064 nm (cal.)
114	$SrGe_2O_3S_2$(*114*)	[GeO_3S]	0.187@1064 nm (cal.)
115	K_2BaGeS_5(*114*)	[GeS_5]	0.192@1064 nm (cal.)
116	$LaGaOS_2$(*115*)	[GaO_2S_2]	0.198@1064 nm (cal.)
		**δ-[MX_4]**	
117	Sb_5O_7I(*116*)	[SbO_4]	0.013@1064 nm (cal.)
118	$Ag_2(TeO_2F_2)$(*117*)	[TeO_2F_2]	0.032@1064 nm (cal.)
119	$Sb_4O_5Cl_2$(*116*)	[SbO_4]	0.060@1064 nm (cal.)
120	$Sb_4O_5Br_2$(*116*)	[SbO_4]	0.077@1064 nm (cal.)
121	$AgTeO_2F$(*117*)	[TeO_3F]	0.078@1064 nm (cal.)
122	$Sb_4O_5I_2$(*116*)	[SbO_4]	0.084@1064 nm (cal.)

123	$BaPbTe_2O_6$(*118*)	[PbO_4]	0.086@590 nm (cal.)
124	$Sb_2(TeO_3)(SO_4)_2$-$P2_1/c$(II)(*96*)	[SbO_4]	0.093@546 nm (cal.)
125	$Zn_4(Te_3O_7)_2(SO_4)_2(H_2O)$(*82*)	[TeO_4]	0.094@532 nm (cal.)
126	$Sb_2(TeO_4)(SO_4)$(*119*)	[TeO_4]	0.113@546 nm (cal.)
127	$Ca_2Sb_2O(SO_4)_4$(*120*)	[SbO_4]	0.114@546 nm (cal.)
128	$Sb_2(TeO_3)(SO_4)_2$-P-1(I)(*96*)	[SbO_4]	0.118@546 nm (cal.)
129	$Ca_2TeV_2O_9$(*121*)	[TeO_4]	0.125@546 nm (cal.)
130	$Sb_4O_3(TeO_3)_2(HSO_4)(OH)$(*122*)	[TeO_4] and [PbO_4]	0.137@546 nm (cal.)
131	$NH_4Sb(SO_4)_2$(*120*)	[SbO_4]	0.150@546 nm (cal.)
132	$KTeOVO_4$(*121*)	[TeO_4]	0.160@546 nm (cal.)
133	$KSb_2C_2O_4F_5$(*123*)	[SbO_2F_2]	0.170@546 nm (cal.)
134	$RbSb(SO_4)_2$(*124*)	[SbO_4]	0.171@546 nm (cal.)
135	SbB_2O_4F(*125*)	[SbO_4]	0.171@1064 nm (cal.)
136	$CsSb(SO_4)_2$(*124*)	[SbO_4]	0.174@546 nm (cal.)
137	$SbHPO_3F$(*126*)	[SbO_3F]	0.180@546 nm (cal.)
138	$Hg_3(Te_3O_8)(SO_4)$(*82*)	[TeO_4]	0.184@532 nm (cal.)
139	β-$BaTeMo_2O_9$(*127*)	[TeO_4]	0.226@435 nm (exp.)
140	$CdTeMoO_6$(*128*)	[TeO_4]	0.287@514 nm (exp.)
141	$Mo(H_2O)Te_2O_7$(*129*)	[TeO_4]	0.528@546 nm (exp.)
		**This work**	
**142**	**[$C(NH_2)_3$][ICl_4]**	**[ICl_4]**	**0.954@550 nm** (cal.)<br>**0.975@550 nm** (exp.)

Data file S1.

Crystallographic information file (CIF) for [$C(NH_2)_3$][ICl_4] (CCDC 2483461). The file contains the crystallographic data and structural parameters obtained from single-crystal X-ray diffraction analysis of [$C(NH_2)_3$][ICl_4]. The crystallographic data have been deposited with the Cambridge Crystallographic Data Centre (CCDC) under deposition number 2483461.

REFERENCES

1. M. F. Weber, C. A. Stover, L. R. Gilbert, T. J. Nevitt, A. J. Ouderkirk, Giant birefringent optics in multilayer polymer mirrors. *Science* **287**, 2451–2456 (2000).

2. A. Tagaya, H. Ohkita, M. Mukoh, R. Sakaguchi, Y. Koike, Compensation of the birefringence of a polymer by a birefringent crystal. *Science* **301**, 812–814 (2003).

3. X. Chen, B. Zhang, F. Zhang, Y. Wang, M. Zhang, Z. Yang, K. R. Poeppelmeier, S. Pan, Designing an excellent deep-ultraviolet birefringent material for light polarization. *J. Am. Chem. Soc.* **140**, 16311–16319 (2018).

4. Y. Sun, L. Wang, Z. Zhu, X. Li, H. Sun, Y. Zhao, C. Peng, J. Liu, S. Zhang, M. Li, A 3D-printed ferromagnetic liquid crystal elastomer with programmed dual-anisotropy and multi-responsiveness. *Adv. Mater.* **35**, e2302824 (2023).

5. J. Yin, J. Guo, H. Huo, X. Liu, X. Cheng, Z. Lin, L. Wu, L. Chen, Enhanced coplanarity and giant birefringence in hydroxypyridinium nitrate via hydrogen bonding between planar donors and planar acceptors. *Angew. Chem. Int. Ed. Engl.* **137**, e202417579 (2025).

6. S. Niu, G. Joe, H. Zhao, Y. Zhou, T. Orvis, H. Huyan, J. Salman, K. Mahalingam, B. Urwin, J. Wu, Giant optical anisotropy in a quasi-one-dimensional crystal. *Nat. Photonics* **12**, 392–396 (2018).

7. A. Tudi, S. Han, Z. Yang, S. Pan, Potential optical functional crystals with large birefringence: Recent advances and future prospects. *Coord. Chem. Rev.* **459**, 214380 (2022).

8. T. Ouyang, Y. Shen, S. Zhao, Accurate design and synthesis of nonlinear optical crystals employing $KBe_2BO_3F_2$ as structural template. *Chin. J. Struct. Chem.* **42**, 100024 (2023).

9. A. Ashkin, G. Boyd, J. I. Dziedzic, R. Smith, A. Ballman, J. Levinstein, K. Nassau, Optically-induced refractive index inhomogeneities in $LiNbO_3$ and $LiTaO_3$. *Appl. Phys. Lett.* **9**, 72–74 (1966).

10. M. J. Dodge, Refractive properties of magnesium fluoride. *Appl. Optics* **23**, 1980–1985 (1984).

11. Z. Guoqing, X. Jun, C. Xingda, Z. Heyu, W. Siting, X. Ke, D. Peizhen, G. Fuxi, Growth and spectrum of a novel birefringent α-BaB_2O_4 crystal. *J. Cryst. Growth* **191**, 517–519 (1998).

12. G. Ghosh, Dispersion-equation coefficients for the refractive index and birefringence of calcite and quartz crystals. *Opt. Commun.* **163**, 95–102 (1999).

13. H. Luo, T. Tkaczyk, E. Dereniak, K. Oka, R. Sampson, High birefringence of the yttrium vanadate crystal in the middle wavelength infrared. *Opt. Lett.* **31**, 616–618 (2006).

14. W. M. Sinton, Birefringence of rutile in the infrared. *J. Opt. Soc. Am.* **51**, 1309–1310 (1961).

15. Y. Zhou, Z. Guo, H. Gu, Y. Li, Y. Song, S. Liu, M. Hong, S. Zhao, J. Luo, A solution-processable natural crystal with giant optical anisotropy for efficient manipulation of light polarization. *Nat. Photonics* **18**, 922–927 (2024).

16. H. Sha, D. Yang, Q. Li, L. Yang, Z. Wang, R. Su, C. He, B. Su, X. Yang, X. Long, Designing a heteroleptic tetrahedral group with an ultrahigh polarizability anisotropy by optimizing the bonding electrons activity and their distribution. *Laser Photonics Rev.* **19**, 2401785 (2025).

17. C. Xie, X. Jiang, B. Liu, G. Guo, BaFS: Birefringence enhanced by the transformation from optical isotropy to anisotropy via interlayer anion substitution. *Small* **21**, e2409705 (2025).

18. X. Hao, Y. Su, C. Lin, M. Luo, L. Wu, R. Wang, X. Wen, N. Ye, $(CN_4H_7)H_2C_3N_3S_3$: Trithiocyanurate exhibiting giant optical anisotropy via weak interaction-mediated functional module alignment. *Inorg. Chem.* **64**, 17427–17435 (2025).

19. Z. Chen, C. Li, X. Wu, J. Lu, Z. Yang, X. Hou, M. Mutailipu, Engineered integration of diverse functional units induces multifunctionality in malonate-borate hybrids. *Aggregate* **6**, e70134 (2025).

20. G. Xu, X. Bai, Z. Yang, J. Han, S. Pan, π-Conjugated cations in phosphates: A pathway to solar-blind UV nonlinear optical crystals with phase-matching. *Angew. Chem. Int. Ed. Engl.* **64**, e202510363 (2025).

21. J. Huang, S. Shu, G. Cai, Review of heteroleptic tetrahedra as birefringent or nonlinear optical motifs. *Cryst. Growth Des.* **22**, 1500–1514 (2022).

22. J. Lu, Y. Lian, L. Xiong, Q. Wu, M. Zhao, K. Shi, L. Chen, L. Wu, How to maximize birefringence and nonlinearity of π-conjugated cyanurates. *J. Am. Chem. Soc.* **141**, 16151–16159 (2019).

23. L. H. Liu, C. L. Hu, Z. Y. Bai, F. F. Yuan, Y. S. Huang, L. Z. Zhang, Z. B. Lin, $2(C_3H_7N_6)^+\cdot 2Cl^-\cdot H_2O$: An ultraviolet nonlinear optical crystal with large birefrigence and strong second-harmonic generation. *Chem. Commun.* **56**, 14657–14660 (2020).

24. H. Jia, D. Xu, Z. Li, M. Arif, Y. Jiang, X. Hou, $(C_3N_6H_7)BF_4\cdot H_2O$ and $(C_3N_6H_7)SO_3CH_3\cdot H_2O$ with large birefringence induced by coplanar π-conjugated $[C_3N_6H_7]^+$ groups. *Inorg. Chem. Front.* **11**, 8331–8338 (2024).

25. D. Dou, Q. Shi, Y. Bai, C. Chen, B. Zhang, Y. Wang, $C_3N_6H_7SO_3NH_2$: Non-π-conjugated tetrahedra decoupling π-conjugated groups achieving large optical anisotropy and wide band gap. *Mater. Chem. Front.* **7**, 5924–5931 (2023).

26. W. Huang, X. Zhang, Y. Li, Y. Zhou, X. Chen, X. Li, F. Wu, M. Hong, J. Luo, S. Zhao, A hybrid halide perovskite birefringent crystal. *Angew. Chem. Int. Ed. Engl.* **61**, e202202746 (2022).

27. D. Dou, C. Wei, B. Zhang, D. Yang, Y. Wang, Ultra-high optical anisotropy with UV transmission achieved by rational arrangement of extended π-conjugated groups. *Angew. Chem. Int. Ed. Engl.* **64**, e202504761 (2025).

28. C. Chen, Y. Li, H. Huang, C. Jin, B. Zhang, K. M. Ok, Giant birefringence enabled by the highly anisotropic linear IX_2^-(X= Cl, Br) building blocks. *Angew. Chem. Int. Ed. Engl.* **64**, e202506625 (2025).

29. Y. Song, Y. Zhang, Y. Li, Z. Bai, W. Huang, K. Wang, S. Zhao, J. Luo, Polar $[NH(CH_2SO_3)_2]^{2-}$ dimers enable the first promising iminodimethanesulfonate with nonlinear optical properties and wide UV transparency. *Chin. J. Chem.* **43**, 1399–1404 (2025).

30. H. Chen, P. Gong, Z. Lin, L. Kang, Can low structural anisotropy produce high optical anisotropy? Anomalous giant optical birefringent effect in PI_4AlI_4 in focus. *J. Am. Chem. Soc.* **147**, 3438–3449 (2025).

31. S. Zhang, L. Dong, B. Xu, H. Chen, H. Huo, F. Liang, R. Wu, P. Gong, Z. Lin, Heteroanionic $[VO_xS_{4-x}]$ groups: Tetrahedral units with large birefringence for mid-infrared nonlinear optical crystals. *Inorg. Chem. Front.* **11**, 5528–5535 (2024).

32. Z. Bai, K. M. Ok, Designing sulfate crystals with strong optical anisotropy through π-conjugated tailoring. *Angew. Chem. Int. Ed. Engl.* **136**, e202315311 (2024).

33. P. Li, C. Hu, Y. Li, J. Mao, F. Kong, $Hg_4(Te_2O_5)(SO_4)$: A giant birefringent sulfate crystal triggered by a highly selective cation. *J. Am. Chem. Soc.* **146**, 7868–7874 (2024).

34. C. D. Cornwell, R. S. Yamasaki, Nuclear quadrupole coupling in the alkali chloroiodides. I. chlorine resonances. *J. Chem. Phys.* **27**, 1060–1067 (1957).

35. F. D. Chattaway, F. L. Garton, XXIV.—Tetrachloroiodides of organic bases. *J. Chem. Soc.* **125**, 183–188 (1924).

36. H. Chang, G. Zhang, K. Chou, A sulfur emission-free route for the synthesis of Mo and Mo_2C via carbothermal reduction of MoS_2. *Metall. Mater. Trans. B* **53**, 96–106 (2022).

37. G. Distefano, M. Guerra, D. Jones, A. Modelli, F. P. Colonna, Experimental and theoretical study of intense shake-up structures in the XPS spectra of nitrobenzenes and nitrosobenzenes. *Chem. Phys.* **52**, 389–398 (1980).

38. M. J. Capitán, J. Álvarez, C. Navio, Study of the electronic structure of electron accepting cyano-films: TCNQ versus TCNE. *Phys. Chem. Chem. Phys.* **20**, 10450–10459 (2018).

39. C. Chen, Y. Li, C. Jin, K. M. Ok, $CsICl_2$: A record-birefringent inorganic crystal with ultrawide infrared transparency enabled by linear interhalogen ICl_2^- units. *J. Am. Chem. Soc.* **147**, 26133–26138 (2025).

40. M. Mutailipu, J. Han, Z. Li, F. Li, J. Li, F. Zhang, X. Long, Z. Yang, S. Pan, Achieving the full-wavelength phase-matching for efficient nonlinear optical frequency conversion in $C(NH_2)_3BF_4$. *Nat. Photonics* **17**, 694–701 (2023).

41. G. M. Sheldrick, Crystal structure refinement with SHELXL. *Acta Crystallogr. C Struct. Chem.* **71**, 3–8 (2015).

42. O. V. Dolomanov, L. J. Bourhis, R. J. Gildea, J. A. Howard, H. Puschmann, OLEX2: A complete structure solution, refinement and analysis program. *J. Appl. Cryst.* **42**, 339–341 (2009).

43. A. Spek, Single-crystal structure validation with the program PLATON. *J. Appl. Cryst.* **36**, 7–13 (2003).

44. M. Segall, P. J. Lindan, M. A. Probert, C. J. Pickard, P. J. Hasnip, S. Clark, M. Payne, First-principles simulation: Ideas, illustrations and the CASTEPcode. *J. Phys. Condens. Matter* **14**, 2717–2744 (2002).

45. S. J. Clark, M. D. Segall, C. J. Pickard, P. J. Hasnip, M. I. Probert, K. Refson, M. C. Payne, First principles methods using CASTEP. *Z. Kristallogr. Cryst. Mater.* **220**, 567–570 (2005).

46. J. P. Perdew, K. Burke, M. Ernzerhof, Generalized gradient approximation made simple. *Phys. Rev. Lett.* **77**, 3865–3868 (1996).

47. X. Zhang, C. Jiang, L. Qi, C. Wu, C. Zhang, Enhancement of optical anisotropy from $[BF_4]$ units by ligand substitution. *J. Organomet. Chem.* **1038**, 123756 (2025).

48. K. Li, A. Tudi, C. Xie, W. Jin, L. Liu, M. Mutailipu, K. Poeppelmeier, F. Zhang, Z. Yang, S. Pan, Anion ratio-directed design of $CaB_4O_5F_4$ and $CaB_6O_8F_4$:$[BO_3]/[BO_2F_2]$ hybridization tailoring deep-ultraviolet nonlinear optical performance. *J. Mater. Chem. C* **13**, 18410–18419 (2025).

49. B. Zhang, G. Shi, Z. Yang, F. Zhang, S. Pan, Fluorooxoborates: Beryllium-free deep-ultraviolet nonlinear optical materials without layered growth. *Angew. Chem. Int. Ed. Engl.* **56**, 3916–3919 (2017).

50. M. Arif, X. Liu, H. Jia, Z. Yang, X. Hou, S. Pan, [$CON_3H_6BF_4$]: An efficient metal-free borate with optimized birefringence schieved by structural design strategy. *Small* **21**, 2500633 (2025).

51. Y. Shen, M. Wu, J. Xiong, Y. Lin, M. Li, $(C_9H_7BrN)BF_4$ and $(C_9H_7BrN)_2SiF_6$: Two birefringent crystals with distinct optical anisotropy regulated by anions. *Cryst. Growth Des.* **25**, 5134–5138 (2025).

52. X. Zhang, L. Guo, B. Zhang, J. Yu, Y. Wang, K. Wu, H.-J. Wang, M.-H. Lee, From silicates to oxonitridosilicates: Improving optical anisotropy for phase-matching as ultraviolet nonlinear optical materials. *Chem. Commun.* **57**, 639–642 (2021).

53. P.-L. Wang, P.-E. Werner, L. Gao, R. K. Harris, D. P. Thompson, Ordering of nitrogen and oxygen in nitrogen-containing melilites $Y_2Si_3O_3N_4$ and $Nd_2Si_{2.5}Al_{0.5}O_{3.5}N_{3.5}$. *J. Mater. Chem.* **7**, 2127–2130 (1997).

54. C. Fang, G. De Wijs, R. De Groot, R. Metselaar, H. Hintzen, G. De With, O/N ordering in $Y_2Si_3O_3N_4$ with the melilite-type structure from first-principles calculations. *Chem. Mater.* **12**, 1071–1075 (2000).

55. H.-W. Wei, X.-M. Wang, H. Jiao, X.-P. Jing, Structural correlation between $Ba_3Si_6O_{12}N_2$ and $Ba_3Si_6O_9N_4$ and the luminescent properties Eu^{2+} doped $Ba_3Si_6O_9N_4$. *J. Alloys Compd.* **726**, 22–29 (2017).

56. F. Stadler, R. Kraut, O. Oeckler, S. Schmid, W. Schnick, Synthese, kristallstruktur und festkörper-nmr-spektroskopische untersuchung des oxonitridosilicates basi6n8o. *Z. Anorg. Allg. Chem.* **631**, 1773–1778 (2005).

57. J. A. Kechele, O. Oeckler, F. Stadler, W. Schnick, Structure elucidation of $BaSi_2O_2N_2$-A host lattice for rare-earth doped luminescent materials in phosphor-converted (pc)-LEDs. *Solid State Sci.* **11**, 537–543 (2009).

58. H. A. Höppe, F. Stadler, O. Oeckler, W. Schnick, $Ca[Si_2O_2N_2]$-A novel layer silicate. *Angew. Chem. Int. Ed. Engl.* **43**, 5540–5542 (2004).

59. K. Wu, Z. Yang, S. Pan, $Na_6Si_3S_8O$: The first example of a sulfide silicate exhibiting unusual tri-polymerized $[Si_3S_8O]^{6-}$ units without S-O bonds. *Dalton Trans.* **46**, 13356–13359 (2017).

60. W. B. Park, S. P. Singh, K.-S. Sohn, Discovery of a phosphor for light emitting diode applications and its structural determination, $Ba(Si, Al)_5(O, N)_8:Eu^{2+}$. *J. Am. Chem. Soc.* **136**, 2363–2373 (2014).

61. J. Sjöberg, G. Helgesson, I. Idrestedt, Refinement of the structure of Si_2N_2O. *Cryst. Struct. Commun.* **47**, 2438–2441 (1991).

62. K. Mishra, B. Deboer, P. Schmidt, I. Osterloh, M. Stephan, V. Eyert, K. Johnson, Electronic structures and nature of host excitation in borates. *Ber. Bunsen. Phys. Chem* **102**, 1772–1782 (1998).

63. S. J. Sedlmaier, D. Weber, W. Schnick, Crystal structure of barium oxonitridophosphate, $Ba_3P_6O_6N_8$. *Z. Kristallogr. Cryst. Matter.* **227**, 1–2 (2012).

64. B. Zhang, G. Han, Y. Wang, X. Chen, Z. Yang, S. Pan, Expanding frontiers of ultraviolet nonlinear optical materials with fluorophosphates. *Chem. Mater.* **30**, 5397–5403 (2018).

65. S. J. Sedlmaier, J. S. Auf Der Günne, W. Schnick, $Sr_3P_6O_6N_8$ - a highly condensed layered phosphate. *Dalton Trans.* , 4081–4084 (2009).

66. S. Wendl, M. Mallmann, P. Strobel, P. J. Schmidt, W. Schnick, Ammonothermal synthesis of Ba_2PO_3N - an oxonitridophosphate with non-condensed PO_3N tetrahedra. *Eur. J. Inorg. Chem.* **2020**, 841–846 (2020).

67. M. Pompetzki, M. Jansen, Crystal structures of $Na_3PO_2S_2 \cdot 11H_2O$ and $Na_3POS_3 \cdot 11H_2O$. *Monatsh. Chem.* **133**, 975–986 (2002).

68. A. Marchuk, P. Schultz, C. Hoch, O. Oeckler, W. Schnick, M_2PO_3N (M= Ca, Sr): Ortho-oxonitridophosphates with β-K_2SO_4 structure type. *Inorg. Chem.* **55**, 974–982 (2016).

69. L. Xiong, J. Chen, J. Lu, C.-Y. Pan, L.-M. Wu, Monofluorophosphates: A new source of deep-ultraviolet nonlinear optical materials. *Chem. Mater.* **30**, 7823–7830 (2018).

70. X. Zhang, L. Kang, P. Gong, Z. Lin, Y. Wu, Nonlinear optical oxythiophosphate approaching the good balance with wide ultraviolet transparency, strong second harmonic effect, and large birefringence. *Angew. Chem. Int. Ed. Engl.* **60**, 6386–6390 (2021).

71. J. Lu, J.-N. Yue, L. Xiong, W.-K. Zhang, L. Chen, L.-M. Wu, Uniform alignment of non-π-conjugated species enhances deep ultraviolet optical nonlinearity. *J. Am. Chem. Soc.* **141**, 8093–8097 (2019).

72. D. Mootz, W. Look, G. Sassmannshausen, Die kristallstruktur von ammonium-diamidothiophosphat. *Z. Anorg. Allg. Chem.* **358**, 282–295 (1968).

73. K. Senevirathne, C. S. Day, M. D. Gross, A. Lachgar, N. Holzwarth, A new crystalline LiPON electrolyte: Synthesis, properties, and electronic structure. *Solid State Ion.* **233**, 95–101 (2013).

74. L. Kang, X. Zhang, F. Liang, Z. Lin, B. Huang, Poly (difluorophosphazene) as the first deep-ultraviolet nonlinear optical polymer: A first-principles prediction. *Angew. Chem. Int. Ed. Engl.* **131**, 10356–10360 (2019).

75. M. Pompetzki, M. Jansen, Sodium monothiophosphate (V): Crystal structure and sodium ionic conductivity. *Z. Anorg. Allg. Chem.* **628**, 641–646 (2002).

76. A. Al-Qawasmeh, N. Holzwarth, $Li_{14}P_2O_3N_6$ and Li_7PN_4: Computational study of two nitrogen rich crystalline LiPON electrolyte materials. *J. Power Sources* **364**, 410–419 (2017).

77. D. Mootz, J. Goldmann, Die kristallstruktur von diammonium-amidothiophosphat. *Acta Crystallogr.* **25**, 1256–1262 (1969).

78. T. Zheng, Q. Wang, J. Ren, L. Cao, L. Huang, D. Gao, J. Bi, G. Zou, Halogen regulation triggers structural transformation from centrosymmetric to noncentrosymmetric switches in tin phosphate halides Sn_2PO_4X (X = F, Cl). *Inorg. Chem. Front.* **9**, 4705–4713 (2022).

79. V. Janickis, K. Marøy, The dihydrogenmonothiophosphate and trihydrogendisulfanediphospho nate anions crystal structures of KH_2SPO_3 and $[Co(en)_2Cl_2]H_3S_2P_2O_6$. *Acta Chem. Scand.* **994**, 461–464 (1994).

80. J. Guo, A. Tudi, S. Han, Z. Yang, S. Pan, Sn_2PO_4I: An excellent birefringent material with giant optical anisotropy in non π-conjugated phosphate. *Angew. Chem. Int. Ed. Engl.* **60**, 24901–24904 (2021).

81. Y. Liu, Y. Liu, Z. Lin, Y. Li, Q. Ding, X. Chen, L. Li, S. Zhao, M. Hong, J. Luo, Nonpolar $Na_{10}Cd(NO_3)_4(SO_3S)_4$ exhibits a large second-harmonic generation. *CCS Chem.* **4**, 526–531 (2022).

82. P.-F. Li, C.-L. Hu, Y.-P. Gong, F. Kong, J.-G. Mao, Hg3(Te3O8)(SO4): A new sulfate tellurite with a novel structure and large birefringence explored from d10metal compounds. *Chem. Commun.* **57**, 7039–7042 (2021).

83. Z. Yu, Q. Ding, Y. Jiang, W. Huang, C. Yang, S. Zhao, J. Luo, $Na_{10}Zn(NO_3)_4(SO_3S)_4$: A nonlinear optical crystal combining inorganic π-conjugated and non-π-conjugated heteroanion groups. *Inorg. Chem. Front.* **11**, 107–113 (2023).

84. S. Ke, H. Fan, C. Lin, N. Ye, M. Luo, Constructing ultraviolet nonlinear optical crystals with large second harmonic generation and short absorption edges by using polar tetrahedral S_2O_3 groups. *Inorg. Chem. Front.* **10**, 2811–2817 (2023).

85. W. Jin, W. Zhang, A. Tudi, L. Wang, X. Zhou, Z. Yang, S. Pan, Fluorine-driven enhancement of birefringence in the fluorooxosulfate: A deep evaluation from a joint experimental and computational study. *Adv. Sci.* **8**, e2003594 (2021).

86. Y. Song, X. Hao, C. Lin, D. Lin, M. Luo, N. Ye, Two tellurium (IV)-based sulfates exhibiting strong second harmonic generation and moderate birefringence as promising ultraviolet nonlinear optical materials. *Inorg. Chem.* **60**, 11412–11418 (2021).

87. D. Zhang, S. J. Rettig, J. Trotter, F. Aubke, Superacid anions: Crystal and molecular structures of oxonium undecafluorodiantimonate (V), $[H_3O][Sb_2F_{11}]$, cesium fluorosulfate, $CsSO_3F$, cesium hydrogen bis (fluorosulfate), $Cs[H(SO_3F)_2]$, cesium tetrakis (fluorosulfato) aurate (III), Cs $[Au(SO_3F)_4]$, cesium hexakis (fluorosulfato) platinate (IV), $Cs_2[Pt(SO_3F)_6]$, and cesium hexakis (fluorosulfato) antimonate (V), $Cs[Sb(SO_3F)_6]$. *Inorg. Chem.* **35**, 6113–6130 (1996).

88. X. Hao, M. Luo, C. Lin, G. Peng, F. Xu, N. Ye, $M(NH_2SO_3)_2$ (M= Sr, Ba): Two deep-ultraviolet transparent sulfamates exhibiting strong second harmonic generation responses and moderate birefringence. *Angew. Chem. Int. Ed. Engl.* **60**, 7621–7625 (2021).

89. X. Wang, J. Lee, Y. Li, Y. Kuk, K. M. Ok, $Cd(NH_2SO_3)_2 \cdot xH_2O$ (x= 0, 2): New sulfamates with a unique coordination environment and reversible phase transitions. *Inorg. Chem. Front.* **10**, 1411–1420 (2023).

90. H. Tian, C. Lin, X. Zhao, F. Xu, C. Wang, N. Ye, M. Luo, $Ba(SO_3CH_3)_2$: A deep-ultraviolet transparent crystal with excellent optical nonlinearity based on a new polar non-π-conjugated nlo building unit $SO_3CH_3^-$. *CCS Chem.* **5**, 2497–2505 (2023).

91. K. O'Sullivan, R. Thompson, J. Trotter, Crystal structure of ammonium fluorosulphate. *J. Chem. Soc. A* **5**, 1814–1817 (1970).

92. Z. Žák, M. Kosička, The crystal structure of lithium fluorosulphate $LiSO_3F$. *Acta Crystallogr.* **34**, 38–40 (1978).

93. Y.-P. Gong, Y.-X. Ma, S.-M. Ying, J.-G. Mao, F. Kong, Two indium sulfate tellurites: Centrosymmetric $In_2(SO_4)(TeO_3)(OH)_2(H_2O)$ and non-centrosymmetric $In_3(SO_4)(TeO_3)_2F_3(H_2O)$. *Inorg. Chem.* **58**, 11155–11163 (2019).

94. H. Tian, N. Ye, M. Luo, Sulfamide: A promising deep-ultraviolet nonlinear optical crystal assembled from polar covalent $[SO_2(NH_2)_2]$ tetrahedra. *Angew. Chem. Int. Ed. Engl.* **61**, e202200395 (2022).

95. P.-F. Li, C.-L. Hu, F. Kong, S.-M. Ying, J.-G. Mao, $Y_2(Te_4O_{10})(SO_4)$: A new sulfate tellurite with a unique Te_4O_{10} polyanion and large birefringence. *Inorg. Chem. Front.* **8**, 164–172 (2021).

96. J. Xue, S. Wang, X. Peng, Z. Lin, X. Dong, L. Cao, L. Huang, G. Zou, Dual lone pair strategy for high birefringence in antimony (III)-based tellurite sulfates with short-wave UV transparency. *Inorg. Chem.* **64**, 2133–2139 (2025).

97. Z. Li, Z. Liang, J. Wan, L. Liu, C. Wu, P. Wang, X. Jiang, Z. Lin, H. Liu, Different p-block elements induce C_3[111] octahedral distortion in titanium to generate an intense nonlinear effect. *Chem. Sci.* **16**, 3329–3335 (2025).

98. M. Monz, H. Jacobs, Kaliumamidotrioxogermanate (IV)-Wasserstoff-Brückenbindungen in $K_3GeO_3NH_2$ und $K_3GeO_3NH_2 \cdot KNH_2$. *Z. Anorg. Allg. Chem.* **621**, 137–142 (1995).

99. J. Guyader, R. Jacquet, J. Malhaire, G. Roult, Y. Laurent, Préparation et structure de KGeON: Un composé à liaison potassium-azote. *Rev. Chim. Minér.* **20**, 863–870 (1983).

100. S. Jaulmes, M. Palazzi, Structure cristalline de $Na_3AsO_2S_2 \cdot 11H_2O$. *Acta Crystallogr.* **32**, 2119–2122 (1976).

101. M.-C. Wang, M.-F. Zhuo, Z. Lin, J.-J. Li, M.-B. Xu, Y.-X. Hu, J.-M. Lian, K.-Z. Du, J. Chen, From poor-active to high performance via heteroleptic tetrahedra engineering: Rational design of non-π-conjugated hybrids for phase-matchable ultraviolet nonlinear optics. *J. Mater. Chem. C* **13**, 11651–11660 (2025).

102. M. Palazzi, Structure cristalline de $Na_3AsO_3S \cdot 7H_2O$. *Acta Crystallogr.* **32**, 516–520 (1976).

103. H.-Y. Wu, C.-L. Hu, M.-B. Xu, Q.-Q. Chen, N. Ma, X.-Y. Huang, K.-Z. Du, J. Chen, From $H_{12}C_4N_2CdI_4$ to $H_{11}C_4N_2CdI_3$: A highly polarizable $CdNI_3$ tetrahedron induced a sharp

enhancement of second harmonic generation response and birefringence. *Chem. Sci.* **14**, 9533–9542 (2023).

104. S. Srinivasa, L. Cartz, J. Jorgensen, J. Labbe, Pressure-induced tetrahedral tilting and deformation in Ge_2N_2O. *J. Appl. Cryst.* **12**, 511–516 (1979).

105. J. Yu, B. Zhang, X. Zhang, Y. Wang, K. Wu, M.-H. Lee, Finding optimal mid-infrared nonlinear optical materials in germanates by first-principles high-throughput screening and experimental verification. *ACS Appl. Mater. Interfaces* **12**, 45023–45035 (2020).

106. B. Krebs, H. Wallstab, Thio-hydroxo anions of germanium-preparation, structure and properties of $Na_2GeS_2(OH)_{2.5}H_2O$. *Z. Anorg. Allg. Chem.* **36**, 1400–1406 (1981).

107. M. Melullis, S. Dehnen, Syntheses, crystal structures, UV-Vis spectra and first NMR spectra of new potassium salts of chalcogenogermanates. *Z. Anorg. Allg. Chem.* **633**, 2159–2167 (2007).

108. R. Wang, F. Liang, X. Liu, Y. Xiao, Q. Liu, X. Zhang, L.-M. Wu, L. Chen, F. Huang, Heteroanionic melilite oxysulfide: A promising infrared nonlinear optical candidate with a strong second-harmonic generation response, sufficient birefringence, and wide bandgap. *ACS Appl. Mater. Interfaces* **14**, 23645–23652 (2022).

109. C. L. Teske, $Ba_2ZnGe_2S_6O$: A new oxide sulfide with tetrahedral framework structure. *Z. Naturforsch. B* **35**, 672–675 (1980).

110. R. Wang, Y. Guo, X. Zhang, Y. Xiao, J. Yao, F. Huang, $Sr_5Ga_8O_3S_{14}$: A nonlinear optical oxysulfide with melilite-derived structure and wide band gap. *Inorg. Chem.* **59**, 9944–9950 (2020).

111. C. L. Teske, Über oxidsulfide mit åkermanitstruktur $CaLaGa_3S_6O$, $SrLaGa_3S_6O$, $La_2ZnGa_2S_6O$ und $Sr_2ZnGe_2S_6O$. *Z. Anorg. Allg. Chem.* **531**, 52–60 (1985).

112. X. Zhang, Y. Xiao, R. Wang, P. Fu, C. Zheng, F. Huang, Synthesis, crystal structures and optical properties of noncentrosymmetric oxysulfides $AeGeS_2O$ (Ae= Sr, Ba). *Dalton Trans.* **48**, 14662–14668 (2019).

113. T. Endo, Y. Doi, M. Wakeshima, K. Suzuki, Y. Matsuo, K. Tezuka, T. Ohtsuki, Y. J. Shan, Y. Hinatsu, Magnetic properties of the melilite-type oxysulfide $Sr_2MnGe_2S_6O$: Magnetic interactions enhanced by anion substitution. *Inorg. Chem.* **56**, 2459–2466 (2017).

114. W. Xie, F. Li, J. Chen, Z. Yang, G. Li, S. Pan, Improved birefringence activated by tetrahedra decorated with a single linear unit. *Angew. Chem. Int. Ed. Engl.* **135**, e202307895 (2023).

115. S. Jaulmes, Oxysulfure de gallium et de lanthane $LaGaOS_2$. *Acta Crystallogr.* **34**, 2610–2612 (1978).

116. J. Guo, X. Zhan, J. Lan, X. Liu, S. Zhao, X. Xu, L.-M. Wu, L. Chen, $Sb_4O_5I_2$: Enhancing birefringence through optimization of Sb/I ratio for alignment of stereochemically active lone pairs. *Inorg. Chem.* **63**, 2217–2223 (2024).

117. B. Zhang, J.-H. Wu, C.-L. Hu, Y.-F. Li, F. Kong, J.-G. Mao, From $AgTeO_2F$ and $Ag_2(TeO_2F_2)$ to $Ag_3F_3(TeF_6)(TeO_2)_{12}$: The first silver tellurite oxyfluorides with linear and nonlinear optical properties. *Inorg. Chem. Front.* **10**, 1328–1337 (2023).

118. Y. Yang, Y. Guo, B. Zhang, T. Wang, Y.-G. Chen, X. Hao, X. Yu, X.-M. Zhang, Lead tellurite crystals $BaPbTe_2O_6$ and $PbVTeO_5F$ with large nonlinear−/linear-optical responses due to active lone pairs and distorted octahedra. *Inorg. Chem.* **61**, 1538–1545 (2022).

119. X.-M. Dong, K. Huang, H.-Y. Zhao, C. He, X.-T. An, Q. Wei, G.-Y. Yang, $Sb_2(TeO_4)(SO_4)$: An anhydrous tellurite sulfate with large birefringence and enhanced thermal stability. *Inorg. Chem.* **64**, 7818–7824 (2025).

120. Y. Lan, H. Luo, L. Wang, L. Huang, L. Cao, X. Dong, G. Zou, Two short-wave UV antimony (III) sulfates exhibiting large birefringence. *Inorg. Chem.* **63**, 2814–2820 (2024).

121. T. Wang, H. Luo, S. Zhang, L. Huang, L. Cao, X. Dong, G. Zou, Enhanced birefringence and excellent thermal stability in two tellurium (IV) vanadates: $KTeOVO_4$ and $Ca_2TeV_2O_9$. *Inorg. Chem.* **64**, 4673–4679 (2025).

122. X.-F. Li, K. Wang, C. He, J.-H. Li, X.-T. An, J. Pan, Q. Wei, G.-M. Wang, G.-Y. Yang, $Sb_4O_3(TeO_3)_2(HSO_4)(OH)$: An antimony tellurite sulfate exhibiting large optical anisotropy activated by lone pair stereoactivity. *Inorg. Chem.* **62**, 7123–7129 (2023).

123. Y. Chen, Z. Chen, Y. Zhou, Y. Li, Y. Liu, Q. Ding, X. Chen, S. Zhao, J. Luo, An antimony (III) fluoride oxalate with large birefringence. *Chemistry* **27**, 4557–4560 (2021).

124. Y. Lan, J. Ren, P. Zhang, X. Dong, L. Huang, L. Cao, D. Gao, G. Zou, $ASb(SO_4)_2$ (A= Rb, Cs): Two short-wave UV antimony sulfates exhibiting large birefringence. *Chin. Chem. Lett.* **35**, 108652 (2024).

125. C. Hu, M. Wu, J. Han, Z. Yang, S. Han, S. Pan, New antimony fluorooxoborates with strong birefringence and unprecedented structural characterisation. *Chem. Commun.* **60**, 2653–2656 (2024).

126. Y. Long, X. Dong, L. Huang, H. Zeng, Z. Lin, G. Zou, $SbHPO_3F$: 2D van der Waals layered phosphite exhibiting large birefringence. *Inorg. Chem.* **61**, 16997–17001 (2022).

127. W. Zhang, X. Tao, C. Zhang, Z. Gao, Y. Zhang, W. Yu, X. Cheng, X. Liu, M. Jiang, Bulk growth and characterization of a novel nonlinear optical crystal $BaTeMo_2O_9$. *Cryst. Growth Des.* **8**, 304–307 (2008).

128. C. Li, X. Tian, Z. Gao, Q. Wu, P. Zhao, Y. Sun, C. Zhang, S. Zhang, D. Cui, X. Tao, Controlled growth of layered acentric $CdTeMoO_6$ single crystals with linear and nonlinear optical properties. *Cryst. Growth Des.* **18**, 3376–3384 (2018).

129. T. Wu, X. Jiang, K. Duanmu, C. Wu, Z. Lin, Z. Huang, M. G. Humphrey, C. Zhang, Giant optical anisotropy in a covalent molybdenum tellurite via oxyanion polymerization. *Adv. Sci.* **11**, e2306670 (2024).
